# Beyond Self-Resistance: ABCF ATPase LmrC Is a Signal-Transducing Component of an Antibiotic-Driven Signaling Cascade Accelerating the Onset of Lincomycin Biosynthesis

**DOI:** 10.1128/mBio.01731-21

**Published:** 2021-09-07

**Authors:** Marketa Koberska, Ludmila Vesela, Vladimir Vimberg, Jakub Lenart, Jana Vesela, Zdenek Kamenik, Jiri Janata, Gabriela Balikova Novotna

**Affiliations:** a Institute of Microbiology, The Czech Academy of Sciences, BIOCEV, Vestec, Czech Republic; b Charles University in Prague, Faculty of Science, Department of Genetics and Microbiology, Prague, Czech Republic; c Institute of Microbiology, The Czech Academy of Sciences, Prague, Czech Republic; McMaster University

**Keywords:** ABCF ATPase, *Streptomyces*, antibiotic biosynthesis, antibiotic-mediated signaling, antibiotic resistance, chemical communication, regulation of gene expression, ribosomal regulation, signal transduction, specialized metabolism

## Abstract

In natural environments, antibiotics are important means of interspecies competition. At subinhibitory concentrations, they act as cues or signals inducing antibiotic production; however, our knowledge of well-documented antibiotic-based sensing systems is limited. Here, for the soil actinobacterium Streptomyces lincolnensis, we describe a fundamentally new ribosome-mediated signaling cascade that accelerates the onset of lincomycin production in response to an external ribosome-targeting antibiotic to synchronize antibiotic production within the population. The entire cascade is encoded in the lincomycin biosynthetic gene cluster (BGC) and consists of three lincomycin resistance proteins in addition to the transcriptional regulator LmbU: a lincomycin transporter (LmrA), a 23S rRNA methyltransferase (LmrB), both of which confer high resistance, and an ATP-binding cassette family F (ABCF) ATPase, LmrC, which confers only moderate resistance but is essential for antibiotic-induced signal transduction. Specifically, antibiotic sensing occurs via ribosome-mediated attenuation, which activates LmrC production in response to lincosamide, streptogramin A, or pleuromutilin antibiotics. Then, ATPase activity of the ribosome-associated LmrC triggers the transcription of *lmbU* and consequently the expression of lincomycin BGC. Finally, the production of LmrC is downregulated by LmrA and LmrB, which reduces the amount of ribosome-bound antibiotic and thus fine-tunes the cascade. We propose that analogous ABCF-mediated signaling systems are relatively common because many ribosome-targeting antibiotic BGCs encode an ABCF protein accompanied by additional resistance protein(s) and transcriptional regulators. Moreover, we revealed that three of the eight coproduced ABCF proteins of *S. lincolnensis* are clindamycin responsive, suggesting that the ABCF-mediated antibiotic signaling may be a widely utilized tool for chemical communication.

## INTRODUCTION

The genus *Streptomyces* and several other related genera of *Actinobacteria* (here referred to as streptomycetes) are filamentous soil and marine bacteria characterized by a remarkably rich specialized metabolism. Specifically, the genomes of streptomycetes contain the highest proportion of biosynthetic gene clusters (BGCs) per Mb among all bacteria (1). BGCs encode the biosynthesis of a wide arsenal of bioactive specialized metabolites, which have applications in various areas, particularly in medicine. For instance, streptomycetes produce two-thirds of the clinically used antibiotics of natural origin. However, the relevant biological roles of specialized metabolites in nature are still under debate. The current concept is that antibiotics are produced as a response to cues from competitors to defend the habitats of the organism, producing them in natural competitive environments (2–5). These cues also involve ribosome-targeting antibiotics, which at subinhibitory concentrations act as elicitors of secondary metabolism (6–8). However, antibiotic-sensing systems common to a group of functionally related but structurally distinct ribosome-targeting antibiotics have not been reported. Streptomycete-derived macrolide, ketolide, lincosamide, and streptogramin antibiotics target the peptidyl transferase center (PTC) of the 50S ribosomal subunit or structures in proximity to it (adjacent A- and P-sites or ribosomal exit tunnel). As a result, all these natural products interfere with proteosynthesis and inhibit bacterial cell growth (for a review, see reference 9). Therefore, apart from truly biosynthetic genes, BGCs of 50S ribosomal subunit-targeting antibiotics also encode regulation elements for timely and coordinated production and resistance mechanisms for self-protection. The regulation typically employs global and/or pleiotropic regulators which direct BGC expression via activation of a pathway-specific regulator (for a review, see reference 10). For resistance, one protein can be sufficient to protect the producing strain (11, 12). However, several mechanisms for self-resistance are often encoded in the BGCs, particularly in those for the biosynthesis of 50S ribosomal subunit-targeting antibiotics (13–17). Specifically, these BGCs often encode antibiotic resistance proteins of the ABCF family and a protein(s) with another resistance mechanism. ABCF proteins are cytosolic ATPases of the ABC superfamily that confer resistance by ribosome protection (18) and not by efflux, which was the hypothesis for a long time. All characterized ABCFs to date act on the ribosome, and their common feature is the ATP-dependent modulation of the peptidyl transferase center (PTC) (reviewed in reference 19). However, the biological function of these proteins is not uniform. Notably, the bacterial ABCFs include not only the antibiotic resistance proteins but also a protein, EttA, involved in translational regulation (20, 21). However, the function of the majority of bacterial ABCFs, which are widely distributed in almost all bacteria, with the highest number per genome (8–11) encoded in actinomycetes remains unknown (22).

**FIG 1:**
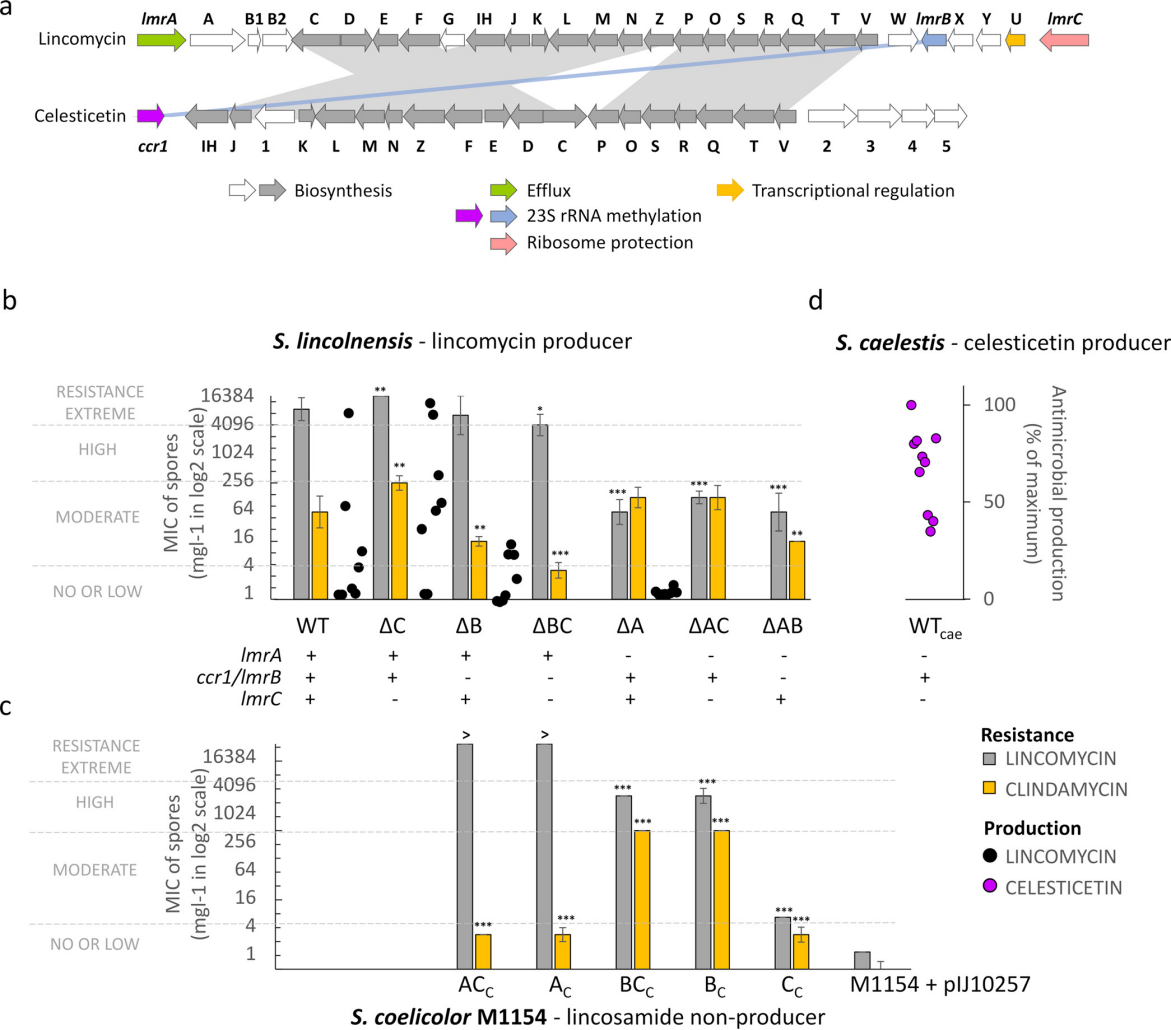
Contribution of resistance proteins to the self-protection and lincomycin production. (a) Lincomycin and celesticetin BGCs share 18 biosynthetic genes encoding the common lincosamide scaffold (in gray) and *lmrB*/*ccr1* resistance genes. Other structural genes encode the specific lincomycin and celesticetin biosynthetic steps (in white), and three remaining lincomycin BGC genes encode the transcriptional activator LmbU and resistance proteins LmrA and LmrC. (b) MIC and lincomycin production of the *S. lincolnensis* ATCC 25466 (WT) and *lmrA* (A), *lmrB* (B), and *lmrC* (C) knockout mutants show that only *lmrA* and *lmrB* are important for the self-protection of *S. lincolnensis*. (c) MICs of S. coelicolor M1154 with an empty vector or with the respective constitutively expressed resistance genes (subscript c). (d) Celesticetin production of the *S. caelestis* ATCC 15084. The MIC values are given as the means ± the SD of *n* ≥ 3 independent measurements. The level of significance of the fold change relative to WT (upper graph) or M1154 (lower graph) is shown (*, *P* < 0.05; **, *P* < 0.01; ***, *P* < 0.001). The “>” sign points to values that exceed 16,384 mg liter^−1^. The levels of lincosamide production for *S. lincolnensis* (*n* = 8) and *S. caelestis* (*n* = 10) are given as a percentage of the maximum production achieved by each strain. The overall table of lincosamide susceptibilities of complemented knockout mutants and MIC values for the mycelia of different growth stages are available in Fig. S1.

In this study, we used the lincomycin BGC as a model BGC encoding three resistance proteins: LmrA, LmrB, and LmrC (Fig. 1a) (23). We show that all Lmr proteins confer a certain level of resistance to lincosamides; however, only the LmrA transporter and partially the LmrB 23S rRNA methyltransferase were required for the self-protection from the produced lincomycin. In contrast and more importantly, we revealed that the LmrC ABCF ATPase is dispensable for self-protection, but it is a key component of an antibiotic-induced cascade, which directs the onset of lincomycin biosynthesis through a transcriptional regulator, LmbU (24, 25), and in cooperation with LmrA and LmrB resistance proteins. The regulation-resistance unit characterized here represents the first reported antibiotic-driven activation of a BGC mediated by an ABCF resistance protein. At the same time, the dual resistance-regulatory function of the LmrC ABCF protein reported here is unprecedented.

10.1128/mBio.01731-21.1 FIG S1 Lincosamide susceptibilities of Streptomyces lincolnensis ATCC 25466 WT, knockout, and complemented strains and Streptomyces coelicolor M1154 carrying *lmrABC* resistance genes on an integrated plasmid. MICs were determined by the agar dilution method inoculated either with spores (*n* = 3 to 6) or with mycelium from the seed (42 h) and production (162 h) cultures (*n* = 2). Resistance genes were cloned under its natural promoter (subscript “n”), and under the constitutive *ermEp* promoter in plasmid pIJ10257 (subscript “c”), or plasmid pIJ6902 (subscript “c2”). Medians of *n* number of replicates and SD of log_2_ values are shown for MICs determined from spores. Download FIG S1, TIF file, 1.8 MB. Copyright © 2021 Koberska et al. 2021 Koberska et al. https://creativecommons.org/licenses/by/4.0/ This content is distributed under the terms of the Creative Commons Attribution 4.0 International license.

## RESULTS

### LmrC antibiotic resistance protein is dispensable for resistance.

To interpret the role of the three resistance proteins encoded in the lincomycin BGC, we first evaluated the contribution of the individual proteins to the resistance. Specifically, we knocked out *lmrA*, *lmrB*, and *lmrC* singly or in pairs in the lincomycin-producing *S. lincolnensis* wild-type (WT) strain (Fig. 1b) and, in addition, we complemented the genes under the control of a constitutive or natural promoter acting in *trans* (see Fig. S1). Furthermore, we constitutively expressed the genes in a lincosamide-sensitive Streptomyces coelicolor M1154 strain (26) (Fig. 1c). Then, we evaluated the resistance phenotype of the WT, knockout, and complemented strains by determining the MICs of lincomycin and its derivative, clindamycin.

We revealed that all strains bearing *lmrA*, including the strains with *lmrA* only (Fig. 1b, *S. lincolnensis* strains WT, ΔC, ΔB, and ΔBC; Fig. 1c, S. coelicolor strains AC_C_ and A_C_), were highly or extremely resistant to lincomycin, while the resistance to lincomycin significantly decreased when *lmrA* was absent regardless of other two resistance genes were present. Interestingly, the resistance to clindamycin, generally a more efficient semisynthetic derivative of lincomycin, is different in this respect. Specifically, the majority of the tested strains were moderately resistant to clindamycin with no or little contribution of LmrA to the resistance (Fig. 1b, compares the strains differing in *lmrA* only: ΔB versus ΔAB, WT versus ΔA, and ΔC versus ΔAC, and Fig. 1c, A_C_ versus M1154+pIJ10257). Therefore, we assume that LmrA, a transporter of the major facilitator family, is highly specific to lincomycin but not clindamycin, and it ensures sufficient self-resistance to the produced lincomycin on its own.

In contrast to the LmrA transporter, the LmrB 23S rRNA monomethyltransferase confers high resistance to lincomycin and clindamycin when overexpressed in S. coelicolor (Fig. 1c, BC_C_ and B_C_). However, when naturally expressed in *S. lincolnensis*, it confers a significant level of resistance to clindamycin alone (Fig. 1b, compares strains differing only in *lmrB*: WT versus ΔB, ΔC versus ΔBC, and ΔA versus ΔAB).

The last resistance protein, LmrC, confers moderate resistance to both lincomycin and clindamycin when overexpressed in S. coelicolor (Fig. 1c, strain C_C_). However, its contribution to the overall resistance in *S. lincolnensis* is not considerable relative to either LmrB (Fig. 1b, compares strains ΔA versus ΔAC) or LmrA (Fig. 1b, compares strains ΔB versus ΔBC). The ΔC knockout strain without *lmrC* showed a slightly increased lincosamide resistance compared to that of the WT (Fig. 1b).

It is worth noting that the complementation of the knockout strains under the control of the putative natural promoter restored the resistance phenotype of the WT except for *lmrB* (see Fig. S1 in the supplemental material). In this case, complementation had to be performed under the control of a constitutive promoter because *lmrB* is cotranscribed with three upstream genes, as evidenced below. Interestingly, constitutive *lmrB* expression resulted in higher resistance values than those of *lmrB* expression in its original genomic context (see Fig. S1). Furthermore, the resistance of *S. lincolnensis* WT and knockout strains was determined from spore suspension, which does necessarily reflect the resistance of the mycelium during lincomycin production. Therefore, we determined the MICs of *S. lincolnensis* deletion strains using spores and mycelia from two different time points of the seed or production cultures (see Fig. S1). Overall, the data for mycelia comply with the data obtained for spores and show that resistance of the mycelium during production increased compared to the mycelium from the seed culture.

Apart from investigating the resistance phenotypes, we determined the amount of lincomycin produced by *S. lincolnensis* WT and single-knockout strains in the culture broth (Fig. 1b). The results support our conclusions drawn from the resistance of the strains. Specifically, the strains with high or extreme resistance to lincomycin were able to produce considerable levels of lincomycin, i.e., the strains bearing both *lmrA* and *lmrB* (which had the largest amount of lincomycin produced) and the strain bearing *lmrA* and not *lmrB* (which had up to 50% of the largest amount of lincomycin produced). On the other hand, strains without *lmrA*, which were the least resistant to lincomycin, produced only traces of lincomycin or nothing.

The dispensability of LmrC for the overall resistance documented above complies with the comparable lincomycin production of ΔC versus WT strains. In addition, the production of lincomycin significantly fluctuated (Fig. 1b). This observation could be explained by a more complex regulation-resistance system (LmbU, LmrA, LmrB, and LmrC) encoded within the lincomycin BGC compared to the highly similar BGC of another lincosamide, celesticetin (12), which contains only one nonbiosynthetic gene, the *ccr1*, coding for Ccr1 23S rRNA monomethyltransferase, as a self-protecting resistance protein homologous to LmrB (Fig. 1a) (for a review, see reference 27). Indeed, the fluctuation of celesticetin produced by *S. caelestis* in parallel cultures is not as pronounced as that of the lincomycin produced by *S. lincolnensis*. Moreover, *S. caelestis* never failed to produce celesticetin (Fig. 1d), while *S. lincolnensis* failed to produce lincomycin in several parallel WT cultures (Fig. 1b).

### Expression of *lmrA*, *lmrB*, *lmrC*, and *lmbU* is induced by clindamycin.

**FIG 2:**
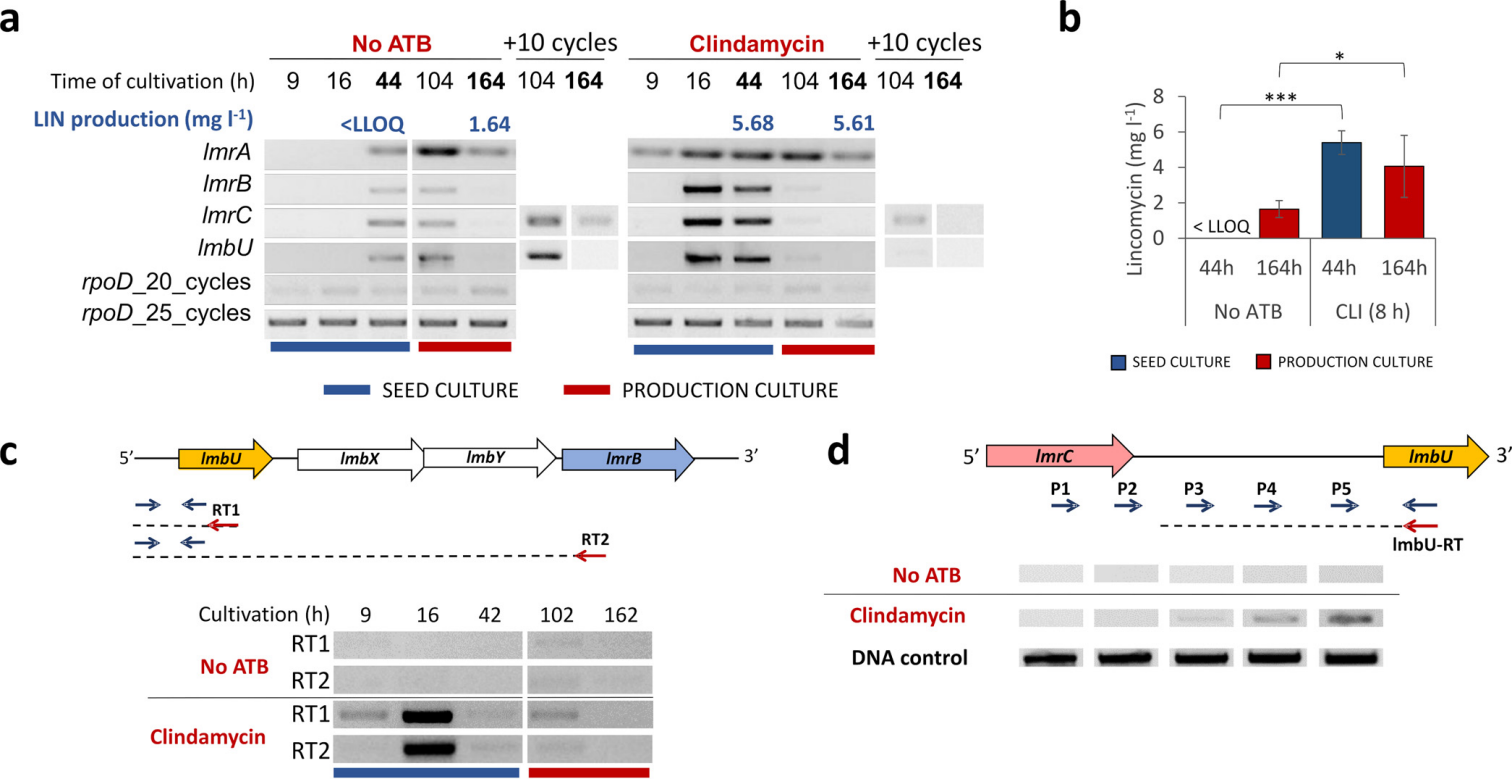
Clindamycin induces the expression of all lincomycin BGC-encoded resistance genes as well as *lmbU* encoding a transcriptional regulator and accelerates lincomycin production. (a) Representative results of RT-PCR and lincomycin LC-MS analyses (*n* = 6) show earlier transcription of *lmrA*, *lmrB*, *lmrC*, and *lmbU* genes and accelerated lincomycin production in *S. lincolnensis* WT after clindamycin supplementation at the eighth hour of culture (0.5 mg liter^−1^). The results of all six independent cultivations are available in Fig. S2. The *rpoD* gene encoding the RNA polymerase sigma factor was used as an internal control. PCR amplification after 25 cycles is shown if not stated otherwise. (b) A summary of lincomycin production levels at 44 and 164 h in six independent cultivations (see Fig. S2) shows that the addition of clindamycin activates lincomycin biosynthesis in the seed culture and increases the total lincomycin levels in the production culture. LLOQ is a lower limit of quantitation (0.031 mg liter^−1^). (c and d) Results of RT-PCR mapping show that *lmrB* is transcribed within the *lmbUXY-lmrB* operon (c) and that *lmrC* is transcribed independently on *lmbU* (d). Representative results of three biological replicates are shown. RT-PCR mapping results for the *lmrC* transcript 3′ end are available in Fig. S3a.

Given our hypothesis of the complex regulation-resistance system of lincomycin production, we wondered whether the expression of any of the *lmbU*, *lmrA*, *lmrB*, and *lmrC* genes could be affected by the produced antibiotic. Therefore, we cultured *S. lincolnensis* WT and divided the culture before the onset of lincomycin biosynthesis into two parallel cultures, one of which was supplemented with clindamycin at a subinhibitory concentration (Fig. 2a). At several time points, we semiquantitatively monitored the expression of the respective genes by reverse transcription-PCR (RT-PCR). Supplementation with clindamycin allowed us to distinguish between the lincosamide used to study its effect on the gene expression and the lincosamide produced by the strain, which we determined by ultrahigh-performance liquid chromatography.

10.1128/mBio.01731-21.2 FIG S2 Transcriptional analysis of *lmrABC* and *lmbU* and lincomycin production in response to clindamycin. RT-PCR and lincomycin LC-MS analyses of six independent cultures showed earlier transcription of *lmrA*, *lmrB*, *lmrC*, and *lmbU* genes and accelerated lincomycin production in *S. lincolnensis* WT after clindamycin supplementation at the eighth hour of culture (0.5 mg liter^−1^). LLOQ is a lower limit of quantitation (0.031 mg liter^−1^). Download FIG S2, TIF file, 3.0 MB. Copyright © 2021 Koberska et al. 2021 Koberska et al. https://creativecommons.org/licenses/by/4.0/ This content is distributed under the terms of the Creative Commons Attribution 4.0 International license.

10.1128/mBio.01731-21.3 FIG S3 RT-PCR analysis of the lmrC-lmbU intergenic region shows that *lmrC* is transcribed independently of *lmbUXY-lmrB* operon. (a) RT-PCR mapping of the *lmrC* 3′ end transcript showed the presence of a transcription terminator immediately downstream of the *lmrC* STOP codon. (b) RT-PCR mapping of the *lmbU* start showed that in *lmrC* knockout (ΔC), *lmbU* is cotranscribed with the apramycin resistance gene. RT-PCR analyses were performed for total RNA isolated from WT and ΔC cultures induced or uninduced by clindamycin (0.5 mg liter^−1^; Fig. S6C). Download FIG S3, TIF file, 0.3 MB. Copyright © 2021 Koberska et al. 2021 Koberska et al. https://creativecommons.org/licenses/by/4.0/ This content is distributed under the terms of the Creative Commons Attribution 4.0 International license.

The results in Fig. 2a show that supplementation with clindamycin induced the expression of all the studied genes—*lmbU*, *lmrB*, *lmrA*, and *lmrC*—in the earlier stages of growth (9 to 16 h) compared to that of the untreated cultures (44 to 104 h), while no effect of clindamycin was observed on the *rpoD* control. In agreement with this observation, the onset of lincomycin production also shifted toward an earlier time of cultivation (44 h) in the cultures supplemented with clindamycin and reached higher values at the end of production culture (Fig. 2b; see also Fig. S2). The transcription of *lmrA* was induced more readily and was detectable over a longer period, while the relative amount of *lmrC*, *lmbU*, and *lmrB* transcripts decreased over time. Similar profiles of *lmrC*, *lmbU*, and *lmrB* transcripts indicate that these genes might be in the same operon. Amplification of *lmbU* from the 1st DNA strand synthesized using a primer specific to *lmrB* demonstrated that the expression of *lmrB* is directly coupled with that of *lmbU* and the two biosynthetic genes *lmbX* and *lmbY* (Fig. 2c). On the other hand, an analogous mapping of the start of *lmbU* transcript (Fig. 2d) and the end of *lmrC* transcript (see Fig. S3a) showed that the *lmrC* gene is transcribed independently of the *lmbUXY-lmrB* operon.

### LmrC is essential for the antibiotic-induced onset of lincomycin production.

**FIG 3:**
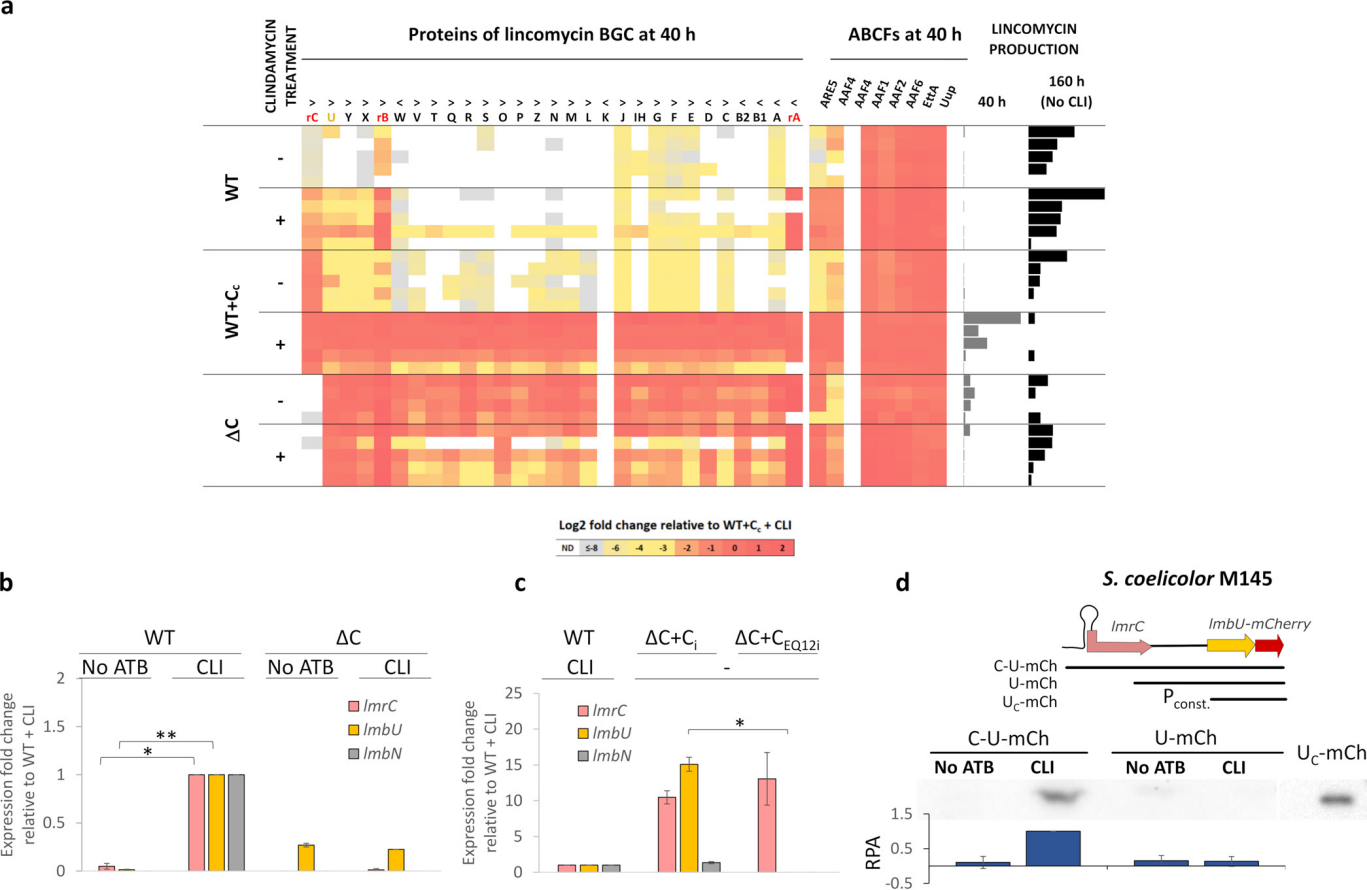
Only ATPase-active LmrC is required for the induction of *lmbU* transcription by clindamycin. (a) Proteomic analysis showing the effect of LmrC and clindamycin on lincomycin biosynthesis and ABCF protein abundance. Ten independent cultures of each *S. lincolnensis* WT, WT constitutively expressing *lmrC* (WT+C_c_), and *lmrC* knockout (ΔC) strain were cultured for 40 h in seed medium with or without clindamycin (CLI) and for another 120 h in production medium without clindamycin. Lincomycin production was quantified after 40 h and at the end of production culture (160 h). Values of lincomycin production are independently expressed as a percentage of maximum production at each time point. Statistical analysis of the proteomic data is shown in Fig. S4a and Table S1. A comparison of WT growth with or without clindamycin supplementation is available in Fig. S4b. Resistance genes (in red) are indicated by the prefix “r.” The orientation of genes in lincomycin BGC are indicated by “less-than” (<) and “more-than” (>) signs. (b and c) Results of the qRT-PCR analysis (*n* = 4) of the WT and ΔC from the 16-h seed culture show that LmrC is required to activate *lmbU* and *lmbN* transcription in response to clindamycin (b) and that only ATPase-active LmrC can activate *lmbU* and *lmbN* transcription (c). The production of LmrC and its ATPase-deficient mutant LmrC_EQ12_ in ΔC were inducible by theophylline (ΔC+C_i_ and ΔC+C_EQ12i_, respectively). The data are expressed relative to the WT cultured with clindamycin; the error bars indicate standard deviation, and the asterisks represent the level of significance (*, *P* < 0.05; **, *P* < 0.01). The evidence that LmrC_EQ12_ does not affect growth is shown in Fig. S4c. (d) Western blot showing the induction of *lmbU* expression by clindamycin-induced LmrC in the heterologous host S. coelicolor M145 with the respective plasmids. U-mCherry (U-mCh) levels in 16-h seed culture mycelium uninduced or induced by clindamycin (0.03 mg liter^−1^) were detected by mCherry-specific antibody in S. coelicolor M145 carrying plasmids C-U-mCh, U-mCh, and U_C_-mCh. A representative Western blot is shown. The graph below shows the average values of relative protein abundancy (RPA) in three independent experiments.

Given the newly defined function of ABCF proteins as modulators of ribosomal PTC, the onset of lincomycin production in response to antibiotics might be regulated by LmrC. To uncover the role of LmrC, we performed comparative mass spectrometry proteomic analysis of the mycelia of *S. lincolnensis* WT, WT+C_c,_ and ΔC strains grown in the absence or presence of clindamycin. As shown in Fig. 3a, clindamycin supplementation increased the abundance of lincomycin BGC proteins in both the WT (namely, proteins of the *lmbUXYB* operon) and WT+C_c_ (the whole BGC), while in the ΔC strain, lincomycin BGC proteins were more abundant in cultures without clindamycin. The induction by clindamycin was also observable at the lincomycin production level at 40 h in the WT+C_c_ strain but not in the ΔC knockout strain, where higher production levels were independent of clindamycin treatment (Fig. 3a). No lincomycin was detected in clindamycin supplemented WT cultures, which contradicts the experiment in Fig. 2a, where high levels of lincomycin were detected at the end of seed culture supplemented with clindamycin. Slight differences in cultivation conditions might be responsible for the shifted onset of lincomycin production between the two experiments (see Fig. S7b). Nevertheless, these results suggest that LmrC is required for the onset of lincomycin biosynthesis triggered by clindamycin. To confirm that LmrC is essential for the transduction of antibiotic signal to the expression of lincomycin BGC, we quantified the transcripts of *lmrC*, *lmbU*, and *lmbN* genes in *S. lincolnensis* WT and ΔC cultured with or without clindamycin at a time point before lincomycin BGC expression. As shown in Fig. 3b, the clindamycin induced transcription of *lmrC*, *lmbU*, and *lmbN*, which was not under the direct control of *lmbU*, in the WT strain but not in the *lmrC-*deficient ΔC knockout strain. Notably, the observed low-level constitutive transcription of *lmbU* in the ΔC strain can be explained by the insertion of apramycin cassette (see Fig. S3b), causing a polar effect. This phenomenon explains the increased production of proteins in ΔC (Fig. 3a). However, it is important for our reasoning that neither protein production nor *lmbU* transcription in the ΔC strain is affected by clindamycin. ABCF family proteins generally exhibit ATPase activity, which is required for protein function (22). Therefore, we wondered whether LmrC is a functional protein capable of ATPase activity that induces gene expression. Hence, we complemented the ΔC knockout strain with *lmrC* or *lmrC_EQ12_* expressed from a theophylline-inducible plasmid (C_i_). The overproduction of functional LmrC resulted in the expression of *lmbU* and *lmbN*, while the overproduction of ATPase-deficient LmrC_EQ12_ mutant did not have this effect (Fig. 3c). Notably, the expression of *lmbU* and *lmbN* mediated by the overproduction of LmrC was achieved without supplementation with clindamycin, and a similar phenomenon was observed at the protein level when LmrC was produced constitutively in the WT (the comparison of WT and WT+C_C_ without clindamycin treatment is shown in Fig. 3a). Altogether, these results demonstrate that clindamycin induces the production of LmrC, which in turn induces the production of LmbU, which is a known activator of lincomycin biosynthesis (24).

10.1128/mBio.01731-21.7 FIG S7 Streptomycete cultivation, antibiotic treatment, and sample collection. (a) MICs determined from spores. (b) *S. lincolnensis* growth for lincomycin production, LIN BGC gene expression, and proteomic analysis. Arrows indicating time of antibiotic supplementation and culture sampling are colored depending on the subsequent analysis: LC-MS lincomycin production in blue, RT-PCR expression analysis in red, nLC-MS^2^ proteomic analysis in green, and Western blot analyses in black. (c) S. coelicolor growth for Western blot analysis of the *lmrC* attenuator. Download FIG S7, TIF file, 0.9 MB. Copyright © 2021 Koberska et al. 2021 Koberska et al. https://creativecommons.org/licenses/by/4.0/ This content is distributed under the terms of the Creative Commons Attribution 4.0 International license.

10.1128/mBio.01731-21.4 FIG S4 Growth and proteomic analysis of *S. lincolnensis* lmrC knockout (ΔC) and overexpression (WT+C_c_) strains. (a) Volcano plots showing the distribution of protein abundance fold change and *P* values (two-tailed *t* test, false discovery rate [FDR] of 0.05, with a fudge factor S0 = 0.1) for each strain induced by clindamycin and for the WT+C_c_ and ΔC compared to the WT. (b) Growth of *S. lincolnensis* WT and ΔC is affected by the presence of a subinhibitory concentration of clindamycin (0.5 mg liter^−1^), while WT+C_c_ is not. (c) The growth of ΔC is affected by the production of the partial ATPase active mutant LmrC E495Q (C_EQ2i_) but not by the ATPase-deficient double mutant LmrC E167Q E495Q (C_EQ12i_). Genes encoding LmrC and its mutants are expressed from the *ermEp* promoter combined with the theophylline-dependent riboswitch (subscript “i”). Fivefold dilutions of spore suspension starting at OD_450_ 0.2 were spotted onto agar without or with 3 mM theophylline and LIN (1 mg liter^−1^). Plates were incubated at 30°C for 5 days. Download FIG S4, TIF file, 1.6 MB. Copyright © 2021 Koberska et al. 2021 Koberska et al. https://creativecommons.org/licenses/by/4.0/ This content is distributed under the terms of the Creative Commons Attribution 4.0 International license.

10.1128/mBio.01731-21.8 TABLE S1 Biosynthetic gene clusters and representative actinomycete genomes used as a source for the phylogenetic tree of ABCF proteins in Fig. 6b. Download Table S1, PDF file, 1.1 MB. Copyright © 2021 Koberska et al. 2021 Koberska et al. https://creativecommons.org/licenses/by/4.0/ This content is distributed under the terms of the Creative Commons Attribution 4.0 International license.

### The antibiotic-LmrC-LmbU signaling cascade is independent of other *S. lincolnensis* regulatory elements.

Several recent studies described regulators of lincomycin biosynthesis encoded outside the BGC in the *S. lincolnensis* genome (28–31). Some of these conserved global regulators might be involved in the antibiotic-induced onset of lincomycin production in addition to LmrC. To rule out this hypothesis, we cloned the lincomycin BGC region starting upstream of *lmrC* and ending with *lmbU* translationally fused with the mCherry reporter (C-U-mCh) and introduced it into S. coelicolor M145. Truncated versions of the construct without the 5′-half *lmrC* (U-mCh) and lmbU-mCherry expressed from the constitutive *ermEp* promoter (U_C_-mCh) were used as controls (Fig. 3d). As expected, the induced production of the mCherry reporter in response to clindamycin was detected only in the strain with the full-length C-U-mCh construct, while in the strain with the truncated *lmrC* gene (U-mCh), the level of mCherry expression did not change after clindamycin induction. From these data, we concluded that *lmbU* expression, and thus the onset of lincomycin production in response to antibiotics, is mainly triggered by *lmrC*. However, we cannot completely exclude the possibility that S. coelicolor homologs of global regulators that contribute to lincomycin biosynthesis in *S. lincolnensis*, such as AdpA and BldD (32, 33), also affect the expression of *lmrC* and *lmbU* in S. coelicolor.

### LmrC production is induced by the LS_A_P group of antibiotics and dampened by LmrA and LmrB.

**FIG 4:**
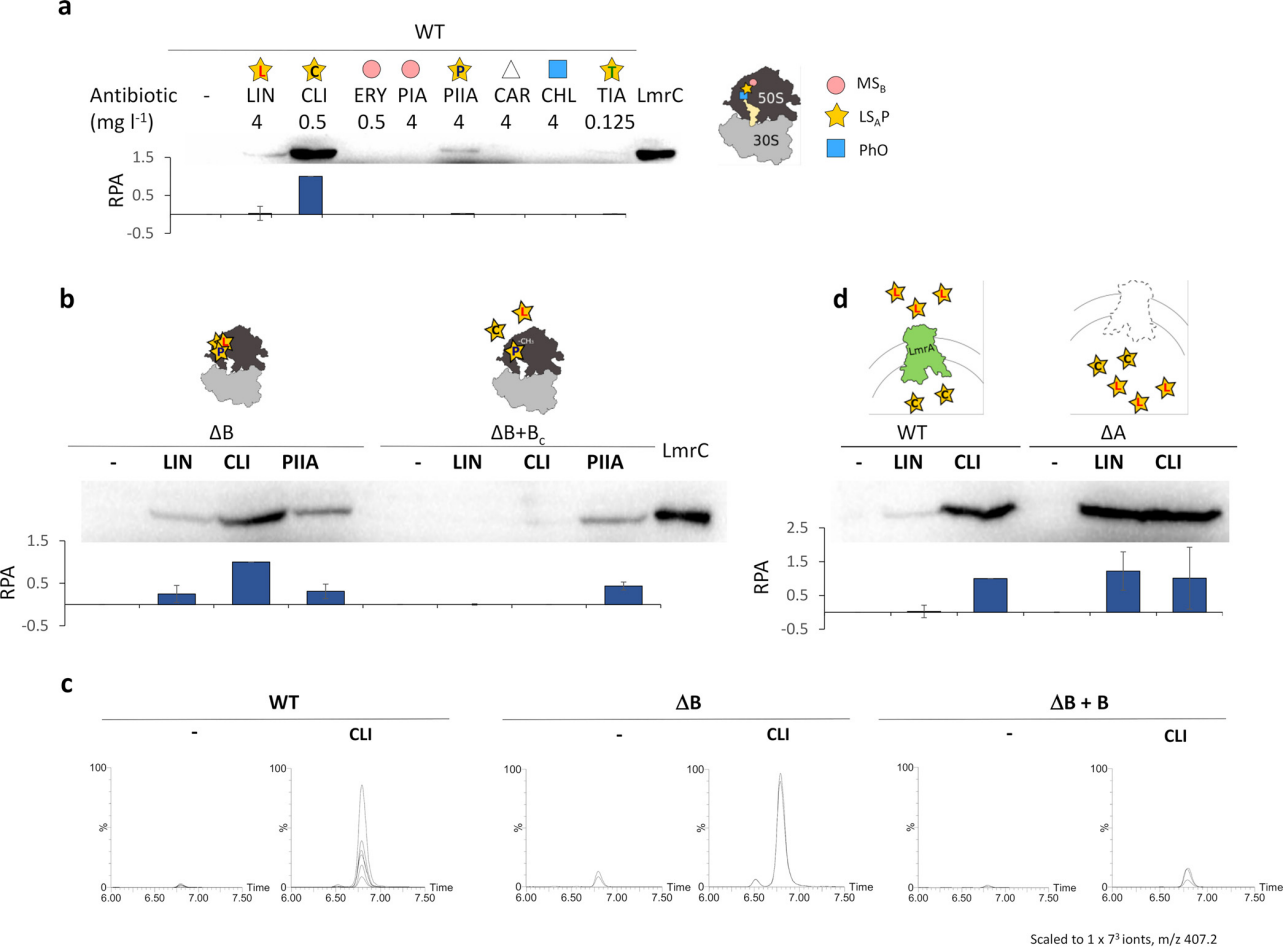
LmrC production and lincomycin biosynthesis are induced by LS_A_P antibiotics and dampened by LmrA and LmrB. Western blot analysis of LmrC production in 16-h seed culture mycelium uninduced or induced by antibiotics. Protein production was detected using an LmrC-specific antibody (see Fig. S5a and b). (a) LmrC levels in the WT after the addition of antibiotics (LIN, lincomycin; CLI, clindamycin; PIIA, pristinamycin IIA; TIA, tiamulin; ERY, erythromycin; PIA, pristinamycin IA; CHL, chloramphenicol; CAR, carbenicillin). A schematic illustration of the overlapping binding sites of the antibiotic groups (represented by colored symbols) defined by resistance phenotypes conferred by antibiotic resistance ABCF proteins (LS_A_P, lincosamides, streptogramins A, pleuromutilins; MS_B_, macrolides, streptogramin B; PhO, phenicol, and oxazolidinones) is shown. (b) LmrC production is not induced by lincomycin and clindamycin in ΔB+B_C_ with constitutive *lmrB* expression because lincosamides cannot bind to the ribosomes methylated by LmrB. In contrast, pristinamycin IIA, which can bind to methylated ribosomes, retained the ability to induce LmrC production. (c) Lincomycin produced in the media of the WT (*n* = 6), *lmrB* knockout (ΔB, *n* = 2), and ΔB+B_C_ (*n* = 3) strains after 42 h of seed culture with or without clindamycin supplementation (see Fig. S7b). (d) LmrC production in response to lincomycin was higher in the *lmrA*-null mutant (ΔA) lacking a specific lincomycin exporter than in the WT. In panels a, b, and d, representative Western blots are shown, and the graphs below show the average values of relative protein abundancy (RPA) in three independent experiments.

We have shown that the LmrC-induced transcription of *lmbU* is largely dependent on how LmrC production is regulated in response to antibiotics. To gain more insight into the regulation of LmrC production, we first tested whether the inducing antibiotics were limited only to clindamycin. For this purpose, we treated *S. lincolnensis* WT cells with a range of ribosome-targeting antibiotics and a cell wall-targeting carbenicillin, and we detected LmrC protein levels using an LmrC-specific antibody. In addition to clindamycin, only lincomycin (lincosamide group), pristinamycin IIA (streptogramin A group), and tiamulin (pleuromutilin group), with lower efficiency, induced LmrC production (Fig. 4a). Interestingly, the activity of these lincosamide-streptogramin A-pleuromutilin (LS_A_P) antibiotics with overlapping binding sites on the ribosome (Fig. 4a) is compromised by antibiotic resistance ABCF proteins as exemplified by Vga(A)_LC_ in staphylococci (18, 34, 35). This suggests that the regulation of LmrC production is coupled to its resistance function, which is the dislocation of the antibiotic from its specific overlapping binding sites within the PTC. Next, we investigated whether the lincomycin BGC-encoded resistance proteins LmrA and LmrB can affect the whole cascade by dampening *lmrC* expression. First, we evaluated LmrC protein levels in *S. lincolnensis* ΔB+B with constitutive overproduction of the LmrB methyltransferase. As shown in Fig. 4b, LmrC production no longer responded to lincomycin and clindamycin, which do not bind to ribosomes methylated by LmrB, but instead remained responsive to the treatment with pristinamycin IIA (streptogramin A group), which can bind to methylated ribosomes (36). Since the *lmrB* gene is in the *lmbUXY-lmrB* operon encoding biosynthetic enzymes (Fig. 2c and d), LmrB is an ideal candidate to provide a feedback loop of the cascade. Indeed, pronounced induction of lincomycin production was apparent in strain ΔB compared to the WT, whereas in the ΔB+B strain, constitutive *lmrB* expression dampened the onset of lincomycin biosynthesis (Fig. 4c).

10.1128/mBio.01731-21.5 FIG S5 Western blot analysis of LmrC levels in *S. lincolnensis* ATCC 25466 and S. coelicolor M1154. (a) Specificity of the LmrC-polyclonal antibody was validated using 16-h seed culture mycelium of *S. lincolnensis* WT and *lmrC* knockout (ΔC) strains grown in the absence or presence of lincomycin (LIN, 4 mg liter^−1^) or clindamycin (CLI, 0.5 mg liter^−1^). (b) Scheme of the constructs used for *lmrC* expression and comparison of LmrC production levels detected by LmrC antibody. The *lmrC gene* was either expressed from its natural promoter (marked with subscript “n”) or under the constitutive *ermEp* promoter (expression from Hyg^r^ vector pIJ10257 marked with subscript “c,” expression from Apr^r^ vector pIJ6902 marked with subscript “c2”). (c) Representative Western blots and analysis of three repetitions showing *S. lincolnensis* WT LmrC protein levels at time points after supplementation with LIN (4 mg liter^−1^) and CLI (4 mg liter^−1^). (d) Clindamycin-inducible production of the LmrC-mCherry reporter in *S. lincolnensis* WT and S. coelicolor M145 strains and the effect of the active or inactive ATPase-deficient mutant *lmrC*_EQ12_ on induction. Download FIG S5, TIF file, 1.3 MB. Copyright © 2021 Koberska et al. 2021 Koberska et al. https://creativecommons.org/licenses/by/4.0/ This content is distributed under the terms of the Creative Commons Attribution 4.0 International license.

Next, we evaluated the effect of LmrA on LmrC protein levels. Since LmrA confers high-level resistance only to lincomycin (Fig. 1b; see also Fig. S1), there was a considerably higher level of LmrC in the WT induced by clindamycin than in the WT induced by lincomycin (Fig. 4a and b), which may reflect the fact that only lincomycin is exported by the LmrA transporter; thus, the low intracellular levels are maintained (37). Indeed, deletion of *lmrA* (ΔA) resulted in comparable levels of LmrC expression induced by either lincomycin or clindamycin (Fig. 4d). LmrA thus specifically dampens the LmrC production induced by lincomycin by reducing its intracellular concentration. Since both LmrB and LmrA reduce *lmrC* expression in response to antibiotics, we propose that alongside their resistance function, they also serve as a negative feedback loop to the antibiotic-LmrC-LmbU signaling cascade of lincomycin biosynthesis.

### LmrC production is regulated by ribosome-mediated transcriptional attenuation.

**FIG 5:**
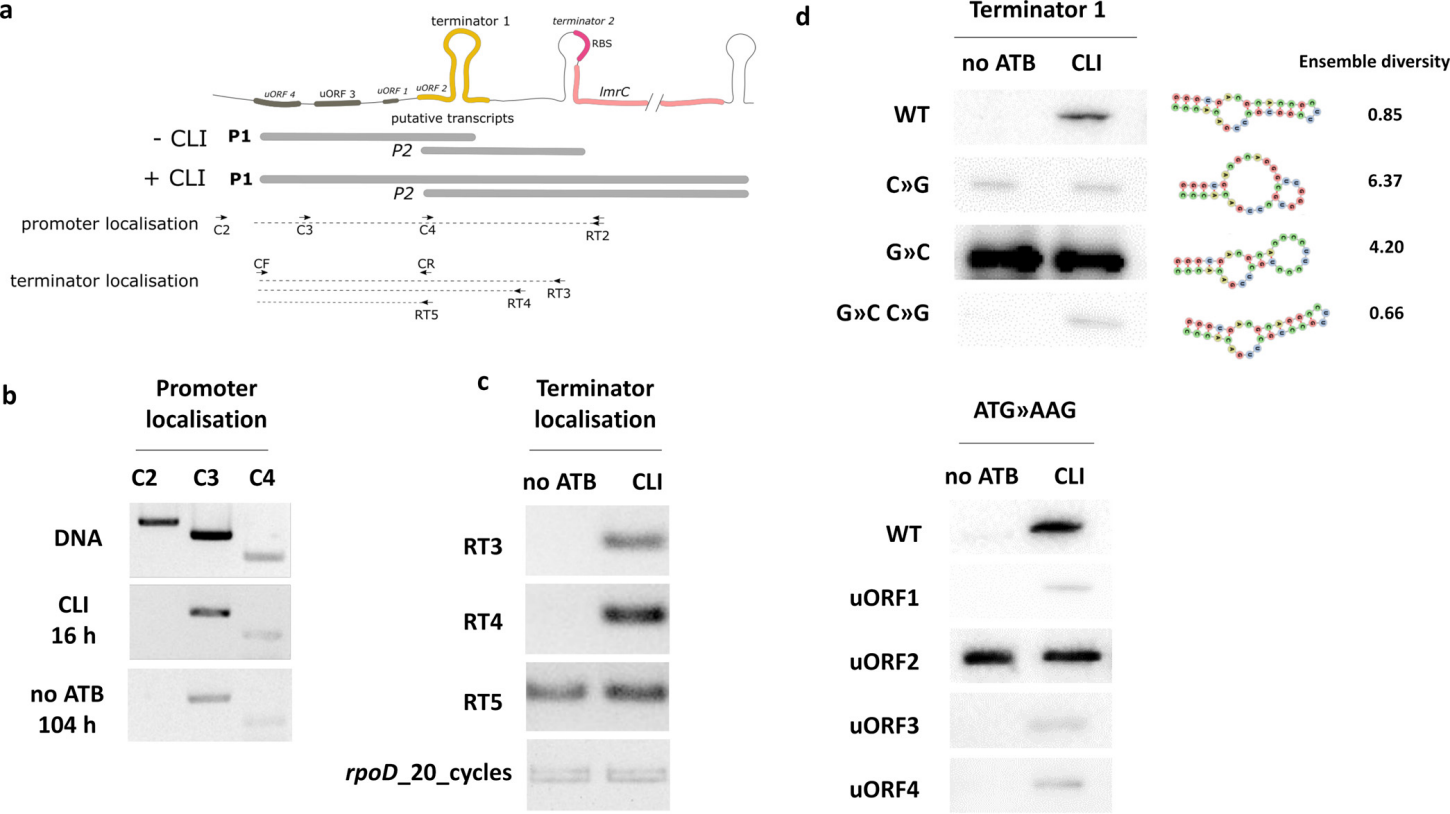
LmrC production is regulated by a ribosome-mediated transcriptional attenuation mechanism. (a) An *in silico* analysis of the lmrC 5′UTR indicates the presence of two promoters predicted by NNPP (74) and G4PromFinder (75) with NNPP probability scores of 0.83 and 0.89 for P1 and P2, respectively, two transcriptional terminators predicted by PASIFIC (Prediction of Alternative Structures for Identification of Cis-regulation) (41) with PASIFIC scores of 0.71 and 0.44 for terminators 1 and 2, respectively, and four putative regulatory uORFs. The RT-PCR mapping strategy used to validate promoter and terminator predictions is shown. (b to d) The results of RT-PCR mapping (*n* = 3) show that *lmrC* is transcribed from the P1 promoter both after the addition of clindamycin (16 h, CLI) (b) and at the natural start of *lmrC* transcription (104 h, –no ATB) (c), *lmrC* transcript is prematurely terminated in the absence of clindamycin in the region between RT primers 4 and 5, thus validating the terminator 1 prediction. (d) Mutational analysis of the 5′UTR of *lmrC* validates the proposed ribosome-mediated attenuation mechanism. Western blot analysis of the LmrC-mCherry (C-mCh) reporter with unmutated (WT) or mutated *lmrC* 5′UTR was performed. Mutations within the predicted terminator disrupted (C»G and G»C) and reconstituted (G»C and C»G) the CLI-inducible production of C-mCh, while the mutagenesis of start codons of the predicted uORFs (ATG»AAG) identified uORF2 to be important for antibiotic-induced *lmrC* expression. The ensemble diversity of the shown secondary structures indicates the average base-pair distance in each mutated terminator loop. The positions of the individual mutations are depicted in attenuator models (see Fig. S6a). Summary data of the Western blot analysis of independent experiments are shown in Fig. S6b.

Substantially reduced production of LmrC in the *lmrB*-overexpression strain (Fig. 4b) after antibiotic induction showed that the binding of the antibiotic to the ribosome is a prerequisite for the induction of LmrC production. LmrC could thus be regulated by a ribosome-mediated attenuation mechanism as described previously for other antibiotic-resistant ABCF proteins (38–40): in the absence of antibiotics, either the formation of a premature terminator in the 5′ untranslated region (5′UTR) or the inaccessibility of ribosome binding site (RBS) prevent gene expression. In the presence of antibiotics, inhibited ribosomes stall during translation of the upstream regulatory open reading frame (uORF), which promotes the alteration of the 5′UTR secondary structure and thereby releases gene expression (41). Indeed, an *in silico* analysis of the *lmrC* upstream region revealed two putative promoters and two premature terminators with the ability to form alternative antiterminator conformations and several short uORFs (Fig. 5a). To examine whether the attenuation mechanism is involved in the control of *lmrC* expression, we first used RT-PCR to map from which of the two predicted promoters *lmrC* is transcribed and the position of the premature terminator (Fig. 5a). The analysis of RNA from the 16-h time point, where *lmrC* is induced by clindamycin, and from the 104-h time point, where *lmrC* transcription starts naturally without clindamycin supplementation (Fig. 2a), showed that in both cases, the *lmrC* transcript starts from promoter P1 (Fig. 5b). As shown in Fig. 5c, the position of the premature terminator was mapped to the region between primers RT 4 and 5, which corresponds to the position of the predicted terminator 1 (Fig. 5a). Next, we prepared a reporter system in which the *lmrC* upstream region, including its promoter, and full-length *lmrC* were translationally fused to mCherry (C-mCh). We introduced the construct into *S. lincolnensis* WT and S. coelicolor M145 strains and determined mCherry levels with or without clindamycin (see Fig. S5d). The mCherry-specific signal was detected only in the presence of clindamycin in both strains, so further experiments were performed in S. coelicolor M145. A series of G-to-C and C-to-G point mutations (see Fig. S6a) in the terminator hairpin led to the disruption and restoration of clindamycin-induced C-mCh production, confirming the terminator prediction (Fig. 5d). To localize the uORF, we mutated the start codons of four upstream ORFs (ATG to ATC or ATG to AAG; Fig. S6a). Surprisingly, only the disruption of uORF2, which partially overlaps with the terminator, led to strong constitutive expression of C-mCh, whereas mutations in other ORFs did not affect C-mCh production (Fig. 5d). This observation suggests an unusual attenuation mechanism in which uORF2 translation is required to form a terminator structure. In summary, the antibiotic-mediated control of *lmrC* expression occurs *via* the formation of a premature terminator structure, which prevents *lmrC* expression in the absence of an antibiotic. LS_A_P antibiotics, if bound to PTC, trigger the shift from the terminator to the antiterminator conformation, enabling *lmrC* transcription (see Fig. S6a). The *lmrC* transcript, specifically its attenuator, is thus the primary sensor of the antibiotic-LmrC-LmbU signaling cascade for lincomycin biosynthesis.

10.1128/mBio.01731-21.6 FIG S6 Mutational analysis of *lmrC* RNA *cis*-regulatory element. (a) Two mutually exclusive structures of putative attenuator 1 were predicted by the PASIFIC algorithm with a score of 0.71. Key regulatory regions include the anti-terminator in orange, terminator in green/blue, and a short uORF of 16 amino acids (yellow) encoded in a region that encompasses the terminator region. The positions of triple G-to-C and C-to-G mutations that were created to disrupt the terminator stem are marked by red and blue arrows, respectively. The positions of point mutations in start codons of putative uORFs are marked by gray and yellow arrows. Mutations in the terminator stem changed the amino acid sequence of uORF2 as indicated. (b) Summary of Western blot analyses (at least three observations for each strain) shows the effect of mutations depicted in Fig. S6a on the clindamycin inducibility of the LmrC-mCherry reporter (C-mCh). The graph shows the log_10_ values of the relative protein abundance (RPA) of each mutated strain with or without clindamycin induction. Download FIG S6, TIF file, 1.2 MB. Copyright © 2021 Koberska et al. 2021 Koberska et al. https://creativecommons.org/licenses/by/4.0/ This content is distributed under the terms of the Creative Commons Attribution 4.0 International license.

### LmrC is coproduced with seven other ABCFs, two of which are responsive to a lincosamide.

The LmrC ABCF protein has a regulatory function, which transduces an antibiotic signal to activate lincomycin biosynthesis in *S. lincolnensis*. Comprehensive phylogenetic analysis classified 30 subfamilies of bacterial ABCF proteins (22). Four subfamilies (Uup, Etta, YdiF, and YbiT) have a broad distribution, while others, including subfamilies with the resistance function (ARE1-7), are taxon specific. *Actinobacteria* is a phylum with the highest number of ABCFs, including seven subfamilies specific to this taxon (AAF1-6, ARE4-5). In addition to LmrC, which belongs to the ARE5 subfamily, the genome of *S. lincolnensis* encodes eight ABCF proteins, three of which have putative resistance activity (ARE5 encoded by SLINC_7152 and two AAF4 encoded by SLINC_1109 and SLINC_6197). We speculated whether some of these resistance proteins are induced by clindamycin and thus could have an antibiotic-responsive regulatory function. We used the same mass spectrometry proteomics data set as used for the comparative analysis of lincomycin biosynthetic proteins to analyze the abundance of ABCF proteins in *S. lincolnensis* WT, WT+C_c_, and ΔC strains grown in the absence or presence of clindamycin. As shown in Fig. 3a, all but one ABCF protein was present in all samples, but only two (ARE5 and AAF4) out of three putative antibiotic-resistant ABCF proteins were substantially upregulated by clindamycin or produced lincomycin. The third putative resistance ABCF protein was not detected in any of the samples. Considering that the putative resistance function of these clindamycin-responsive ABCF proteins is redundant, they may have a regulatory function similar to LmrC.

## DISCUSSION

Antibiotic resistance proteins associated with BGCs have traditionally been perceived as a means of self-protecting mechanisms. It has been proposed that the expression of multiple resistance genes within the same BGC is regulated to optimize the self-protective resistance levels at different stages of growth or biosynthesis to minimize the fitness cost of the resistance expression (42) or to synchronize the resistance in sibling cells (43, 44).

**FIG 6:**
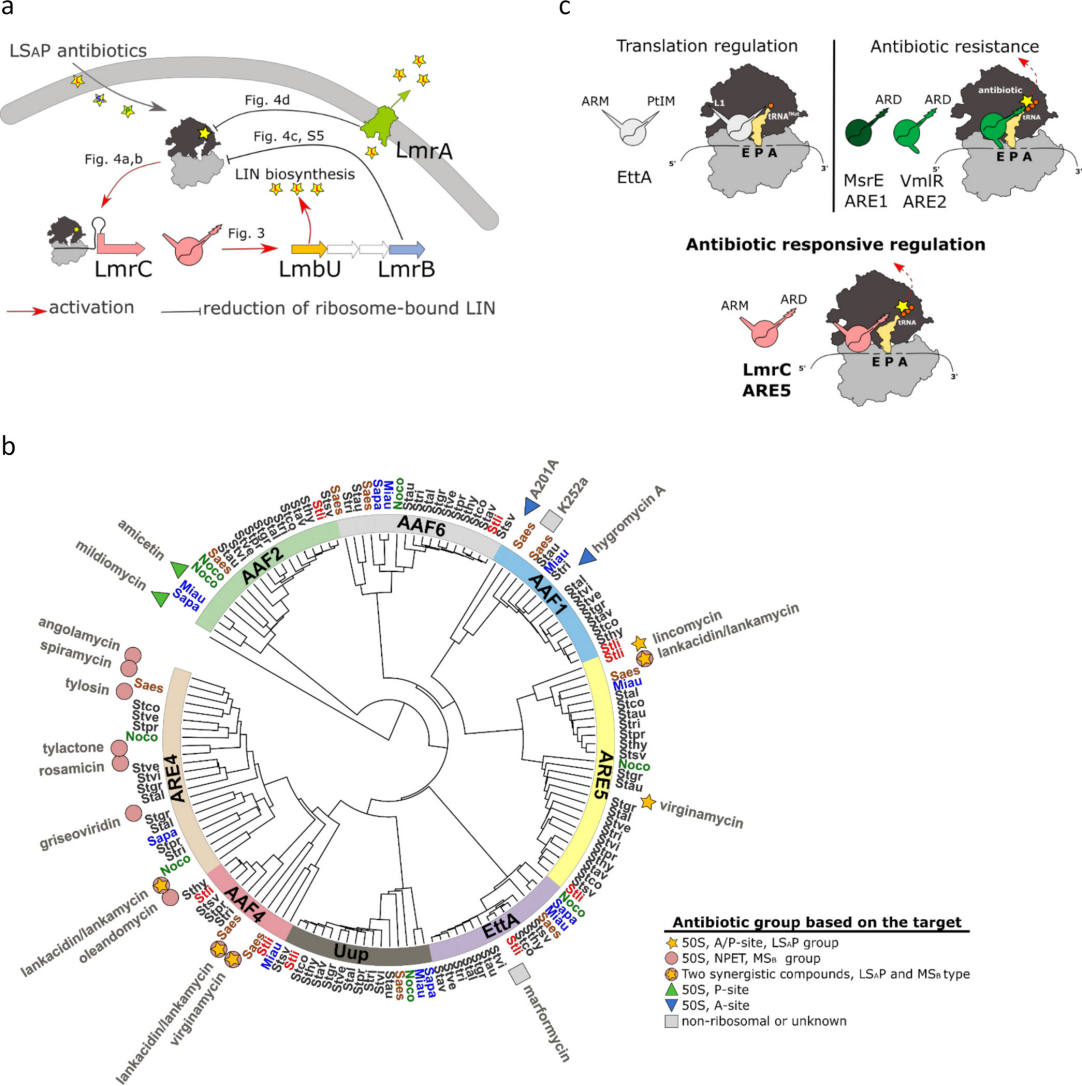
ABCF proteins encoded in biosynthetic gene clusters are putative regulators of antibiotic production in response to antibiotics that share a ribosomal binding site. (a) Scheme of the antibiotic-LmrC-LmbU signaling cascade identified in this study. The production of the LmrC protein is induced by ribosome-bound LS_A_P antibiotics via a ribosome-mediated attenuation mechanism, and it is coordinated with the LmrB and LmrA resistance proteins, which individually reduce the amount of ribosome-bound antibiotic. LmrC then transduces the antibiotic signal from the ribosome to the transcription of *lmbU*. The LmbU transcriptional regulator activates the expression of subordinate biosynthetic genes (24). (b) Phylogenetic tree of ABCF proteins from 14 representative streptomycete genomes and ABCF proteins from previously characterized BGCs. ABCF proteins from characterized BGCs are marked with the name of produced antibiotic: symbols in the legend indicate the antibiotic group. Note the correlation between the antibiotic group and the ABCF subfamily. The genomic ABCF protein sequences were taken from previously published data (22). A list of streptomycete genomes and BGCs is available in Table S1. (c) LmrC domain architecture combines features of resistance and regulatory ABCF proteins. The presence of the arm domain, resembling the ABCF translation regulator EttA, indicates the regulatory function of LmrC, while the antibiotic resistance domain (ARD) is shared with other structurally characterized ABCF resistance proteins. The peptidyl tRNA interaction motif (PtIM) in EttA and ARD structural motifs refers to a linker that separates two ATP binding domains. The ARD domain is significantly longer than PtIM, allowing direct interaction with PTC.

In this study, we characterized an LS_A_P antibiotic-driven signaling cascade for the activation of the onset of lincomycin biosynthesis, in which an antibiotic resistance protein, LmrC, from the ARE5 subfamily of ABCF proteins is the key signal-transducing element (Fig. 6a). The mechanism lies in the induction of *lmrC* transcription by ribosome-mediated attenuation, which means that *lmrC*, specifically its attenuator-forming upstream 5′UTR transcript, is a sensor of LS_A_P antibiotics. Ribosome-mediated attenuation is a common mechanism of regulation of antibiotic resistance ABCF genes in *Firmicutes* (38–40, 45). However, we describe here for the first time its function as a sensor of the signaling cascade. The major novelty of this cascade lies in the dual antibiotic resistance and regulatory function of the ABCF protein LmrC, which transduces the antibiotic signal to the expression of LmbU and promotes lincomycin biosynthesis. In addition, we show that another two lincomycin BGC-encoded resistance proteins, LmrB and LmrA, affect the cascade by dampening the LS_A_P antibiotic-induced expression of *lmrC*. We assume that LmrB, due to its position in the *lmbUXY-lmrB* operon with two biosynthetic genes, mediates a direct negative feedback loop of the cascade. The LmrA transporter links lincomycin biosynthesis to the primary metabolic pathways since it is regulated by the GlnR global regulator (37). LmrA seems to be the most important component for lincomycin production because lincomycin biosynthesis is remarkably suppressed when LmrA is not present. Furthermore, LmrA, as a lincomycin-specific transporter, desensitizes the cascade specifically to lincomycin, which may prevent the products from reactivation the biosynthesis when it is no longer desirable. In addition, the active export of lincomycin contributes to the propagation of antibiotics within the population.

The last component of the regulation cascade, LmbU, is a transcriptional regulator of the newly proposed LmbU family (24). The *lmbU* gene has been evolutionarily accepted along with genes encoding the biosynthesis of the unusual precursor 4-alkyl-l-proline (27), which is a building block of lincomycin and other natural products from *Streptomyces* (46–48). On the other hand, the LmbU homolog is missing in the closely related BGC for the lincosamide celesticetin, which contains proteinogenic l-proline instead of 4-alkyl-l-proline in its structure.

The regulatory pair of LmbU and LmrC is unique to lincomycin BGC; no other known BGC encodes a LmbU-family regulator together with an ABCF protein. On the other hand, BGC-associated ABCF proteins were almost exclusively present in the BGCs for PTC-targeting antibiotics (Fig. 6b; see also Table S1). Most of these BGCs encode additional resistance determinants and pathway-specific transcriptional regulators; however, none are homologous to LmbU (see Table S1). We hypothesize that BGC-encoded ABCF proteins employ transcriptional regulators of various families to form a signaling cascade to activate the biosynthesis of ribosome-binding antimicrobials.

It was previously shown that LmbU directly activates only the 4-alkyl-l-proline biosynthesis-encoding part of lincomycin BGC (24), which is also evident from our proteomic data (Fig. 3a). In contrast, the recently described regulator of lincomycin BGC, AdpA_lin_, activates the entire lincomycin BGC independently of the external lincosamide and thus appears to be the principal regulator of lincomycin biosynthesis (28). What would then be the purpose of the LmrC-LmbU signaling cascade discovered here? This signaling cascade may, in response to the extracellular lincomycin secreted by neighboring cells, induce a premature onset of lincomycin production to ensure its synchronous biosynthesis in a wider population, thereby achieving an ecologically efficient lincomycin concentration similar to that proposed for the biosynthesis of the cell wall inhibiting lantibiotic planosporicin (49) or actinorhodin (50). In addition, we have also shown that the LmrC-LmbU signaling cascade and thus lincomycin production might be activated by functionally similar LS_A_P antibiotics produced by other organisms. Thus, analogous ABCF signaling cascades (Fig. 6b) could coordinate the production of the same types of antibiotics across different organisms sharing a single niche and so mediate cooperative interspecies interactions (51). In support of this concept, a recent study showed that antibiotic production is more likely to be induced by closely related strains or strains sharing BGCs (5). These observations also imply that the ability of antibiotics to induce their own synthesis is a relatively widespread but mostly undetected phenomenon because antibiotic production in the presence of cognate or similar antibiotics is not usually examined.

The induction of specialized metabolism by antibiotics targeting the 50S subunit of the ribosome has been described previously (6, 7, 52), but this is the first time the mechanism of antibiotic sensing and signal transduction has been revealed. The detection of antibiotics by a ribosome *via* a 5′UTR attenuator upstream of the ABCF-encoding gene differs fundamentally from known antibiotic signaling cascades, in which antibiotic or biosynthetic intermediates are detected regardless of their mode of action, typically by direct binding to a transcription factor or its cognate receptor (53). In addition, several examples of resistance systems consisting of antibiotic efflux and cognate, TetR-like transcriptional repressors, such as in the biosynthesis of simocyclinone (54), actinorhodin (50), or landomycin A (55), have been described to promote antibiotic production by sensing a final product or intermediate. The regulatory effect in these examples is facilitated by the export of antibiotics, which is required for high production (50, 54), or is mediated by a cognate antibiotic-recognizing repressor that, in addition to the regulation of transporter, also regulates biosynthetic genes (55). In contrast, LmrC appears to directly transduce the antibiotic signal to lmbU transcription while conferring low antibiotic resistance. However, the exact mechanism of LmrC-driven signal transduction remains to be elucidated. Thus, LmrC has a dual function: resistance and regulation, but it is also possible that the low LmrC-mediated resistance is only an indirect consequence of the primary antibiotic signal transduction function and that its biological significance is minor. Notably, LmrC, as well as other ABCF proteins implicated in antibiotic resistance in streptomycetes, shares the antibiotic resistance domain ARD with structurally characterized antibiotic resistance ABCF proteins, VmlR, MsrE, VgaA_LC_, VgaL, and LsaA from *Firmicutes*. The ARD interacts with PTC to dislodge the antibiotic from the ribosome (34, 35, 56, 57) (Fig. 6c), and it is present in the majority of antibiotic-resistant ABCF proteins but not in EttA and other putative regulatory ABCFs (22). In addition to ARD, LmrC also has the arm domain, which is absent in antibiotic-resistance ABCFs but is present in the EttA translation regulator (20). In EttA, the arm domain restricts ribosome dynamics in response to a lack of available ATP (21). However, further research will be needed to determine whether all the ABCF proteins structurally similar to LmrC, i.e., having both the ARD and the arm domain, have regulatory rather than resistance functions. In addition to LmrC, another two ABCF proteins were induced by clindamycin in *S. lincolnensis* (Fig. 3a), which is a strong indication that ABCF proteins not associated with BGCs for PTC-targeting antibiotics may also have an antibiotic-responsive regulatory function.

The signaling pathway described here, in which the antibiotic signal is sensed and transduced by the dual, resistance, and regulatory ABCF proteins and tuned by two other resistance proteins, points out the need to reconsider the role of antibiotic resistance ABCF proteins as purely protective mechanisms. This discovery also brings together two functionally inconsistent groups of ABCF proteins, antibiotic resistance and regulatory proteins (22, 58), which fundamentally changes the view of these translational ATPases. In addition, given the number of small molecules targeting the 50S ribosomal subunit and the number of bacterial ABCFs encoded by soil bacteria from the *Terrabacteria* group, which includes *Firmicutes* and *Actinobacteria* with the highest number of ABCFs per genome, ABCF-mediated signaling could be one of the most important tools of chemical communication in general.

## MATERIALS AND METHODS

### Bacterial strains and growth conditions.

The strains, plasmids, and oligonucleotides used in this study are listed in Table S2. *Streptomyces* strains were grown at 30°C on solid MS medium (59) (mannitol soya flour medium), DNA (2.3% Difco nutrient agar), and MH agar (1.5% agar in Mueller-Hinton broth, purchased from Oxoid) or in liquid YPM2 (0.4% yeast extract, 0.5% peptone, 1% malt extract [pH 7.2]) or AVM (60) media. The spore suspensions of *S. lincolnensis* were prepared and germinated for 3 h in 2× GM (1% yeast extract, 1% Casamino Acids, 0.01 M CaCl_2_), and the spore suspensions of S. coelicolor were germinated for 6 h in 2× YT (1.6% Bacto tryptone, 1% yeast extract, 0.5% NaCl) according to protocols published in *Practical Streptomyces Genetics* (61). For the selection of exconjugants, antibiotics were added to the cultivation media at the following concentrations: apramycin, 50 mg liter^−1^; kanamycin, 50 mg liter^−1^; carbenicillin, 100 mg liter^−1^; chloramphenicol, 25 mg liter^−1^; nalidixic acid, 25 mg liter^−1^; and hygromycin at 100 mg liter^−1^ (E. coli) or 40 mg liter^−1^ (*Streptomyces*) in agar plates or at 80 mg liter^−1^ (E. coli) or 40 mg liter^−1^ (*Streptomyces*) in liquid media.

10.1128/mBio.01731-21.9 TABLE S2 Statistics for proteomics data (Fig. 3a; see also Fig. S4a). Download Table S2, XLSX file, 0.03 MB. Copyright © 2021 Koberska et al. 2021 Koberska et al. https://creativecommons.org/licenses/by/4.0/ This content is distributed under the terms of the Creative Commons Attribution 4.0 International license.

(i) For antibiotic production, proteomic analysis and RT-PCR, *S. lincolnensis* and its derived mutants or *S. caelestis* spores were germinated and inoculated into 50 ml of YPM2 (to reach optical density at 450 nm [OD_450_] 0.03) in 250-ml baffled flasks and cultivated on an orbital shaker for 40 to 42 h at 30°C and 200 rpm. A total of 2.5 ml of the YPM2 seed culture was used to inoculate 47.5 ml of fresh AVM broth (60), followed by cultivation in 250-ml baffled flasks for 120 h at 30°C and 200 rpm (see Fig. S7b). (ii) For the qPCR analyses and Western blot analyses of *S. lincolnensis*, spores were germinated, inoculated into 50-ml Falcon tubes containing 20 ml of YPM2 media (to reach OD_450_ 0.03), and grown on an orbital shaker (8 h, 30°C, 200 rpm) prior to induction. The cultures were then induced with the antibiotic indicated, and the cultivation continued for an additional 8 h (see Fig. S7b). (iii) For the Western blot analysis of S. coelicolor, spores were germinated, transferred into 50 ml of YEME (0.3% yeast extract, 0.05% peptone, 0.03% malt extract, 1% glucose, 34% sucrose, 5 mM MgCl_2_) in 250-ml flasks with springs, and cultivated on an orbital shaker for 48 h at 30°C and 200 rpm. The cultures were then induced with clindamycin (0.03 mg/liter) and cultivated for 2 h (see Fig. S7c).

### Construction of knockout strains.

The *S. lincolnensis* ΔA (BN3024), *S. lincolnensis* ΔB (BN3002), and *S. lincolnensis* ΔC (BN3001) mutants were constructed by replacing the entire coding sequence of the target gene with a cassette (773 or 775 [62]) carrying the apramycin resistance gene [*aac(3)IV*] and *oriT* of RK2 using the PCR-targeting method (63). Oligonucleotides used for gene deletion and verification of the deletion are listed in Table S2. PCR targeting was applied to the cosmid LK6 (23), which contained the entire lincomycin biosynthetic cluster. After conjugation of mutated cosmids into *S. lincolnensis*, kanamycin-sensitive (Kan^s^) and apramycin-resistant (Apr^r^) double-crossover mutants with target genes replaced by the *aac(3)IV-oriT* cassette were confirmed by PCR amplification. For the construction of *S. lincolnensis* ΔAB (BN3021), *S. lincolnensis* ΔAC (BN3018), and *S. lincolnensis* ΔBC (BN3008) double mutants, the inactivation cassettes 773 in *S. lincolnensis* ΔB (BN3002) and *S. lincolnensis* ΔC (BN3001) single mutants were replaced by an unmarked in-frame deletion obtained by FLP-mediated excision of the disruption cassette (62). The second gene to be deleted was replaced with cassette 775 according to the same protocol as used for single-knockout strains. Knockout strains were verified by PCR and Southern blot analysis. The scheme of the orientation of inactivation cassettes in all knockout strains is available in Fig. S1.

### Construction of vectors for natural, constitutive, or inducible expression.

Details on the preparation of vectors and oligonucleotides used are listed in Table S2. The vectors A_n_ (bearing lmrA with its 1,330-bp upstream sequence), Bn (bearing lmrB gene with an 86-bp upstream region), and Cn (bearing lmrC gene and its 1,281-bp upstream region) were used to express resistance genes under its natural promoter. For constitutive expression, *lmrA*, *lmrB*, and *lmrC* were PCR amplified from ligated under the *ermEp* promoter of pIJ10257 (64), yielding plasmids A_C_, B_C_, and C_C_. For the coexpression of *lmrA* or *lmrB* with *lmrC* in the heterologous host, *lmrC* with the *ermEp* promoter was cloned into PtipA expression vector pIJ6902 (65) (construct C_c2_). All the above-mentioned vectors were prepared by restriction enzyme cloning. Constructs C_C_-mCh, C-U-mCh, U-mCh, U_C_-mCh, 5′C-mCh, and C-mCh were prepared by using the SLICE cloning method (66) (for details, see Table S2). Inducible expression in vectors C_i_ and C_EQ12i_ were achieved by introducing the theophylline-dependent riboswitch (67) via the whole plasmid PCR (details in Table S2). All the constructs described were verified by sequencing.

### Site-directed mutagenesis.

To introduce mutations, the QuikChange protocol (Agilent) was used. In plasmid C_EQ12_, for expression of the lmrC_EQ12_ mutant, two mutations were introduced into the *lmrC* coding sequence: glutamate 167 was replaced with a codon for glutamine, and the parallel codon for glutamate 495 was replaced with a codon for glutamine. To test the putative terminator structure, a series of G-to-C and C-to-G point mutations were introduced into the *lmrC* upstream region in C-mCh vector, yielding plasmids pGBN120, pGBN121, and pGBN124. To test the function of uORFs 1 to 4, their START codons were mutated in C-mCh, yielding plasmids pGBN070 (uORF 1), pGBN106 (uORF 2), pGBN117 (uORF 3), and pGBN119 (uORF 4). All used oligonucleotides are listed in Table S2. All the constructs described were verified by sequencing.

### Antibiotic susceptibility tests.

The MIC values were determined on MH agar with a serial 2-fold dilution of antibiotics. Frozen spores (see Fig. S7a) or mycelia from 42-h seed culture or 120-h production culture (see Fig. S7b) were diluted in 2 ml of sterile water to optical density OD_450_ 0.2 to 0.3, and 5 μl was spotted on MH agar with antibiotic and incubated at 30°C for 5 days.

### Extraction of lincomycin and celesticetin.

A total of 1 ml of supernatant from 42-h seed culture or 160-h production culture (see Fig. S7b) was used for solid-phase extraction as follows: an Oasis HLB 3-ml 60-mg cartridge (hydrophilic-lipophilic balanced sorbent; Waters, USA) was conditioned with 3 ml of methanol and equilibrated with 3 ml of water, and then 1 ml of the supernatant of cultivation broth (for lincomycin extraction pH adjusted to 9.0 with ammonium hydroxide) was loaded. The cartridge was washed with 3 ml of water, and absorbed substances were eluted with 1.5 ml of 80% methanol. The eluent was evaporated to dryness, reconstituted in 150 μl of 50% methanol, and centrifuged at 12,045 × *g* for 5 min at room temperature. The extract was then diluted 10× with methanol-water (1:1 [vol/vol]) and analyzed by liquid chromatography-mass spectrometry (LC-MS), as described below.

### LC-MS analysis of lincomycin and celesticetin.

LC analyses of the samples depicted in Fig. 1 and in Fig. 3a were performed on an Acquity UPLC system equipped with a 2996 DAD detector and LCT premier XE time-of-flight mass spectrometer (Waters). Five microliters of each sample was loaded onto an Acquity UPLC CSH C_18_ LC column (50 mm × 2.1 mm, inner diameter [ID]; particle size, 1.7 μm; Waters) kept at 40°C and eluted with a two-component mobile phase. For phases A and B, the A solution was 1 mM ammonium formate (pH 9) for lincomycin detection (prepared by titration of formic acid 98 to 100% [Merck, Germany] with ammonium hydroxide 28 to 30% [Sigma-Aldrich, Germany]), the A solution was 0.1% formic acid for celesticetin detection, and the B solution was acetonitrile (LC-MS grade; Biosolve, Netherlands). The analyses were performed with a linear gradient program (min/%B): 0/5, 1.5/5, and 12.5/58, followed by a 1.5-min column cleanup (100% B) and 1.5-min equilibration (5% B) at a flow rate of 0.4 ml min^−1^. The DAD detector monitored the column effluent in the range 194 to 600 nm; the mass spectrometer operated in the “W” mode with its capillary voltage set at +2,800 V, its cone voltage at +40 V, its desolvation gas temperature at 350°C, an ion source block temperature at 120°C, cone gas flow at 50 liters h^−1^, desolvation gas flow at 800 liters h^−1^, a scan time at 0.15 s, and an interscan delay at 0.01 s. The data were processed by MassLynx V4.1 (Waters). UV chromatograms monitored at 194 nm were used for lincomycin quantitation based on a five-point linear calibration curve, which was constructed from peak areas corresponding to lincomycin. Calibration solutions were prepared by spiking lincomycin authentic standard at the required concentration into lincomycin-free cultivation broth, extracted and preconcentrated as described above. The quantitation parameters were as follows: concentrations used for the calibration curve were 3.78, 7.56, 15.125, 31.250, 62.5, and 125 mg liter^−1^, the correlation coefficient was *r*^2^ = 0.995, and the limit of quantification was 7.56 mg liter^−1^ (determined as the lowest point of the calibration curve with precision within 10%). Samples from 42 h of cultivation with lincomycin concentrations below the limit of quantitation were examined by MS detection: extracted ion chromatograms at *m/z* 407.2 were evaluated for the presence of lincomycin. The 160-h samples for celesticetin production were also examined using MS detection: extracted ion chromatograms at *m/z* 528.6 were evaluated for the presence of celesticetin.

The LC-MS analyses depicted in Fig. 2b and Fig. S2 were performed on a 6546 LC/Q-TOF (Agilent Technologies, USA) connected to a 1290 Infinity II LC system. One microliter of the sample was loaded on a UPLC CSH C_18_ Premier column (100 mm × 2.1 mm, ID; particle size, 1.7 μm) kept at 30°C. The analytes were eluted at a flow rate of 0.4 ml min^−1^ with a two-component mobile phase consisting of 1 mM ammonium formate (pH 9) (A) and acetonitrile (B) using a linear gradient program min/%B: 0/5, 1.5/5, 15/65, 15.1/100, 16/100, 16.1/5, and 17.5/5. The mass spectrometer operated in ESI+ mode (jet stream technology) with the following settings: capillary voltage, 3,500 V; nozzle voltage, 200 V; gas temperature, 250°C; drying gas, 8 liters min^−1^; nebulizer, 35 lb/in^2^; sheath gas temperature, 400°C; sheath gas flow, 12 liters min^−1^; fragmentor, 140 V; and skimmer, 65 V. The ions of *m/z* 80 to 1,200 were monitored with scan rates of 4 spectra s^−1^ and 250 ms/spectrum. The identity of the analytes was confirmed by the comparison of retention times with an authentic standard, accurate mass, and collision-induced dissociation fragmentation at a collision energy of 20 eV. The data were processed using Quantitative 10.1 software within a MassHunter workstation (Agilent). Lincomycin quantitation was performed using a standard calibration curve of the lincomycin standard (2-fold serial dilutions from 0.0097 to 40 mg liter^−1^) dissolved in the solid-phase extract of a lincomycin nonproducing Streptomyces lincolnensis *lmbD* deletion mutant strain. The LLOQ (lower limit of quantification) was determined as the lowest analyte concentration determined with sufficient precision (relative standard deviation of 20%) and accuracy (80 to 120%) using a calibration curve with its lowest point being equal to LLOQ.

### Protein digestion for proteomic analysis.

Mycelia (0.1 g) of 40-h seed culture inoculated from fresh spores (see Fig. S7b) were lysed in 0.5 ml of 100 mM triethylammonium bicarbonate buffer (pH 8.5) containing 2% sodium deoxycholate, 10 mM Tris(2-carboxyethyl)phosphine, and 40 mM chloroacetamide and boiled at 95°C for 5 min. Protein concentration was determined using a BCA protein assay kit (Thermo), and 20 μg of protein per sample was used for MS sample preparation. Samples were digested with trypsin (at a trypsin/protein ratio of 1/20) at 37°C overnight. After digestion, the samples were acidified with trifluoroacetic acid (TFA) to a final concentration of 1%. Sodium deoxycholate was removed by extraction to ethyl acetate (68), and peptides were desalted on a Michrom C_18_ column.

### nLC-MS^2^ analysis.

Nanoreversed-phase columns (EASY-Spray column, 50 cm by 75 μm, ID; PepMap C_18_; 2-μm particles, 100-Å pore size) were used for LC-MS analysis. Mobile-phase buffer A was composed of water and 0.1% formic acid. Mobile-phase buffer B was composed of acetonitrile and 0.1% formic acid. Samples were loaded onto the trap column (Acclaim PepMap300; C_18_, 5 μm; 300-Å Wide Pore; 300 μm × 5 mm; five cartridges) for 4 min at 17.5 μl min^−1^ loading buffer composed of water, 2% acetonitrile, and 0.1% TFA. Peptides were eluted with a mobile-phase B gradient from 4 to 35% B in 60 min. Eluting peptide cations were converted to gas-phase ions by electrospray ionization and analyzed on a Thermo Orbitrap Fusion (Q-OT-qIT; Thermo). Survey scans of peptide precursors from 350 to 1,400 *m/z* were performed at 120 K resolution (at 200 *m/z*) with a 5 × 10^5^ ion count target. Tandem MS was performed by isolation at 1.5 *m/z* with the quadrupole, higher-energy C-trap dissociation fragmentation with a normalized collision energy of 30, and rapid scan MS analysis in the ion trap. The MS2 ion count target was set to 10^4^, and the maximum injection time was 35 ms. Only those precursors with charge states of 2 to 6 were sampled for MS2. The dynamic exclusion duration was set to 45 s with a 10-ppm tolerance around the selected precursor and its isotopes. Monoisotopic precursor selection was turned on. The instrument was run in the top speed mode with 2-s cycles (69).

### Proteomic data analysis and interpretation.

All data were analyzed and quantified with MaxQuant software (version 1.6.1.0) (70). The false discovery rate (FDR) was set to 1% for both proteins and peptides, and we specified a minimum peptide length of seven amino acids. The Andromeda search engine was used for the MS/MS spectra search against the Streptomyces lincolnensis database (downloaded from the NCBI on July 2018). Enzyme specificity was set with the C terminus as Arg and Lys, also allowing cleavage at proline bonds and a maximum of two missed cleavages. Carbamidomethylation of cysteine was selected as a fixed modification, and N-terminal protein acetylation and methionine oxidation were selected as variable modifications. The “match between runs” feature of MaxQuant was used to transfer identifications to other LC-MS/MS runs based on their masses and retention time (maximum deviation, 0.7 min), and this was also used in quantification experiments. Quantifications were performed with the label-free algorithms described recently. The obtained normalized data were imported to Perseus 1.6.1.3 software (Max Planck Institute of Biochemistry, Munich, Germany) (71). All numeric values corresponding to protein intensity were transformed to a logarithmic scale, and all samples were grouped using categorical annotation. Missing values were then replaced by random numbers drawn from a normal distribution of 1.8 standard deviations (SD) downshift and with a width of 0.3 of each sample. Heat maps of the relative abundance of selected proteins were generated from the matrix of protein intensities without imputation of missing values in Microsoft Excel. Proteomic analysis at 40 h was assessed in five biological (four for ΔC without clindamycin) replicates for each sample/treatment.

### RT-PCR.

Mycelia from 16-h seed cultures were uninduced or induced by clindamycin (0.5 mg liter^−1^) (see Fig. S7c), and 5-ml portions the culture were harvested by centrifugation (4,000 × *g*, 15 min, 4°C) and flash-frozen in liquid nitrogen. For the analysis, samples were defrosted and incubated in 1 ml of RNAprotect cell reagent (Qiagen; 5 min, 25°C). Subsequently, the cells were centrifuged (4,000 × *g*, 15 min, 4°C), and the pellet was resuspended in 250 μl of TE buffer. The suspension was mixed with glass beads (0.1-mm diameter) in a 2:1 ratio and disrupted using a Fast-Prep (MP Biomedicals) program for 1 × 60 s at a speed of 6 ms^−1^. Immediately after cell lysis, total RNA was isolated using TRI Reagent (T9424, 100 ml; Sigma) according to the manufacturer’s protocol. Isolated total RNA, resuspended in 100 μl of water, was treated with Turbo DNase (Invitrogen) according to the manufacturer’s protocol, followed by an additional step of total RNA isolation using TRI Reagent. The integrity of RNA was controlled by 2% agarose gel electrophoresis. The purity and concentration of RNA were controlled by using NanoDrop.cDNA was synthesized using SuperScript III reverse transcriptase (Invitrogen) according to the manufacturer’s protocol. One microliter of reverse transcription reaction mix or total RNA was added to 20 μl of PCR mix using the primers indicated (sequences in Table S2). PCR was performed using Taq-Purple DNA polymerase (T107). The following PCR program was used: 96°C for 1 min, followed by 30 cycles of 96°C for 10 s, 55°C for 20 s, and 72°C for 1 min.

### Quantitative RT-PCR.

The 16-h clindamycin-induced and uninduced seed cultures were cultivated and incubated in 1 ml of Protect RNA in the same manner as for RT-PCR. Total RNA was extracted with an RNeasy RNA isolation kit (Qiagen). The isolated RNA was treated with DNase I (0.1 U μl^−1^, 30 min, 37°C) and repurified with an RNeasy RNA isolation kit. The RNA quantity and quality were checked with a NanoDrop instrument (DeNovix). The RNA quantities were normalized to the lowest concentration of RNA in the samples. The quantities of *lmrC*, *lmbU*, *lmbN*, and *16S rRNA* transcripts were measured by one-step qRT-PCR (SuperScript III Platinum SYBR Green One-Step qRT-PCR kit) using the following oligonucleotides (10 μM): lmrCf+lmrCr, lmbUf+lmbUr, lmbNf+lmbNr, and 16SrRNAf+16SrRNAr. The following real-time PCR program was used: 60°C for 3 min, 95°C for 5 min, followed by 40 cycles of 95°C for 10 s, 65°C (*lmbU*, *lmbN*, and *16S rRNA*) or 63°C (*lmrC*) for 20 s. *C_T_* values of *lmrC*, *lmbU*, and *lmbN* transcripts, based on the standard curves, were normalized to *C_T_* values of 16S rRNA. The relative expression was calculated as 2^–Δ^*^CT^*.

### Western blotting and immunodetection.

For *S. lincolnensis*, 20-ml seed cultures inoculated with germinated spores were induced with lincomycin (4 mg liter^−1^), clindamycin (0.5 mg liter^−1^), pristinamycin IIA (4 mg liter^−1^), tiamulin (0.125 mg liter^−1^), erythromycin (0.5 mg liter^−1^), pristinamycin IA (4 mg liter^−1^), chloramphenicol (4 mg liter^−1^), and carbenicillin (4 mg liter^−1^) at 8 h, and cultivation continued until the indicated time points (see Fig. S7c). Mycelia were harvested by centrifugation (10 min, 4°C, 4,000 × *g*), washed with buffer 1 (50 mM Tris-HCl, 150 mM NaCl [pH 8.0]) and resuspended in sonication buffer (50 mM Tris-HCl, 1× protease inhibitor cocktail [Roche]; pH 8.0). After sonication (3 × 40 s; UP200S Hielscher Ultrasonic GmbH), cell lysates were separated by 8% SDS-PAGE stained with Coomassie blue to test the load of whole protein volume. Next, 8% SDS-PAGE with adjusted whole protein volumes was run, and samples were Western blotted onto a polyvinylidene difluoride membrane (semidry transfer, 15 V, 45 min, Trans-blot; Bio-Rad). A control SDS-PAGE was run in parallel. The membranes were incubated overnight at 4°C in blocking solution 5% (wt/vol) Blotting-Grade Blocker (Bio-Rad) in PBS-Tween buffer (1× PBS, 0.05% Tween 20), followed by incubation for an additional 1 h with primary antibody (rabbit polyclonal anti-LmrC or rabbit polyclonal anti-mCherry antibody) diluted 1:5,000 in 1% (wt/vol) nonfat dried milk in PBS-Tween buffer. Membranes were washed for three times for 15 min each time in PBS-Tween buffer, incubated for 1 h with horseradish peroxidase (HRP)-conjugated monoclonal anti-rabbit IgG 1:2,000 in 1% (wt/vol) Blotting-Grade Blocker in PBS-Tween buffer, and washed three times for 15 min each time in PBS-Tween buffer. Antibody complexes were detected using Immobilon Western HRP substrate (Merck) on a ChemiDoc MP (Bio-Rad). For S. coelicolor, the method was adapted as follows: 50-ml seed cultures inoculated with germinated spores were induced with clindamycin (0.03 mg liter^−1^) at 24 h, and cultivation continued for 2 h. Mycelia were harvested by centrifugation (10 min, 4°C, 4,000 × *g*) and resuspended in lysis buffer (50 mM Tris-HCl, 1× protease inhibitor cocktail [Roche], 1% SDS [pH 8.0]). After lysis on Fast prep (four times for 20 s each time, with 4-min pauses on ice), cell lysates were separated by 10% SDS-PAGE and Western blotted onto a polyvinylidene difluoride membrane (wet transfer, 30 V, 0.09 mA, 18 h). The membranes were incubated for 1 h in blocking solution composed of 5% (wt/vol) Blotting-Grade Blocker (Bio-Rad) in PBS-Tween buffer (1× PBS, 0.05% Tween 20) and for 1 h with primary antibody (rabbit polyclonal anti-mCherry antibody [Invitrogen] or mouse polyclonal anti-mCherry antibody [AB Clonal]) diluted 1:5,000 in 1% (wt/vol) nonfat dried milk in PBS-Tween buffer. Membranes were washed three times for 15 min each time in PBS-Tween buffer, incubated for 1 h with HRP-conjugated monoclonal anti-rabbit or anti-mouse IgG 1:2,000 in 1% (wt/vol) Blotting-Grade Blocker in PBS-Tween buffer, and washed three times for 15 min each time in PBS-Tween buffer. Antibody complexes were detected using Immobilon Western HRP substrate (Merck) on a ChemiDoc MP (Bio-Rad). After immunodetection, the membrane was washed with PBS-Tween buffer and then stained with 0.1% Coomassie R-250 in methanol/water (1:1) for 5 min, destained twice for 10 min each time in acetic acid-ethanol-water (1:5:4), washed with water, and air dried. The dry membrane was recorded on ChemiDoc MP (Bio-Rad) at 600 dpi, and the staining density for each complete lane was analyzed in ImageLab (Bio-Rad) or ImageJ software with an area outside the protein lanes defining the background. The signal intensities were normalized to the signal intensity of the induced BN3038 strain (for S. coelicolor) or BN3511 (for *S. lincolnensis*) transferred to the same membrane.

### Statistical analysis.

The MIC, Western blot, and qRT-PCR results are expressed as the means ± the SD. Differences between two groups were analyzed using a two-sample *t* test (*, *P* < 0.05; **, *P* < 0.01; ***, *P* < 0.001; and *P* > 0.05 if not indicated). Each experiment was performed at least in triplicate unless otherwise stated.

### Antibodies.

The anti-LmrC(2) antibody was generated by GenScript USA, Inc., by inoculating a New Zealand rabbit host strain with a peptide-KLH conjugate containing the LmrC peptide CLQRQAQESAGRAAS. The specificity of the LmrC antibody was validated using *S. lincolnensis* WT and ΔC mycelium from 16-h seed cultures (see Fig. S7c) grown in the absence or presence of lincomycin (LIN; 4 mg liter^−1^) or clindamycin (CLI; 0.5 mg liter^−1^) (see Fig. S5a). The anti-m-Cherry antibody was purchased from Invitrogen (Cat PA5-34974) and AB Clonal. The ribosomal protein S7-specific antibody was obtained from Mee-Ngan F. Yap (72).

### Data availability.

The mass spectrometry proteomics data have been deposited at the ProteomeXchange Consortium via the PRIDE (73) partner repository with the data set identifier PXD026093.

10.1128/mBio.01731-21.10 TABLE S3 Strains, plasmids, and oligonucleotides used in this study (Excel file). Download Table S3, XLSX file, 0.03 MB. Copyright © 2021 Koberska et al. 2021 Koberska et al. https://creativecommons.org/licenses/by/4.0/ This content is distributed under the terms of the Creative Commons Attribution 4.0 International license.
