# Supplementary figures and images for: Beyond Self-Resistance: ABCF ATPase LmrC Is a Signal-Transducing Component of an Antibiotic-Driven Signaling Cascade Accelerating the Onset of Lincomycin Biosynthesis
Source: mBio. 2021 Sep 7;12(5):e01731-21. doi: 10.1128/mBio.01731-21 (PMC8546547; doi:10.1128/mBio.01731-21)

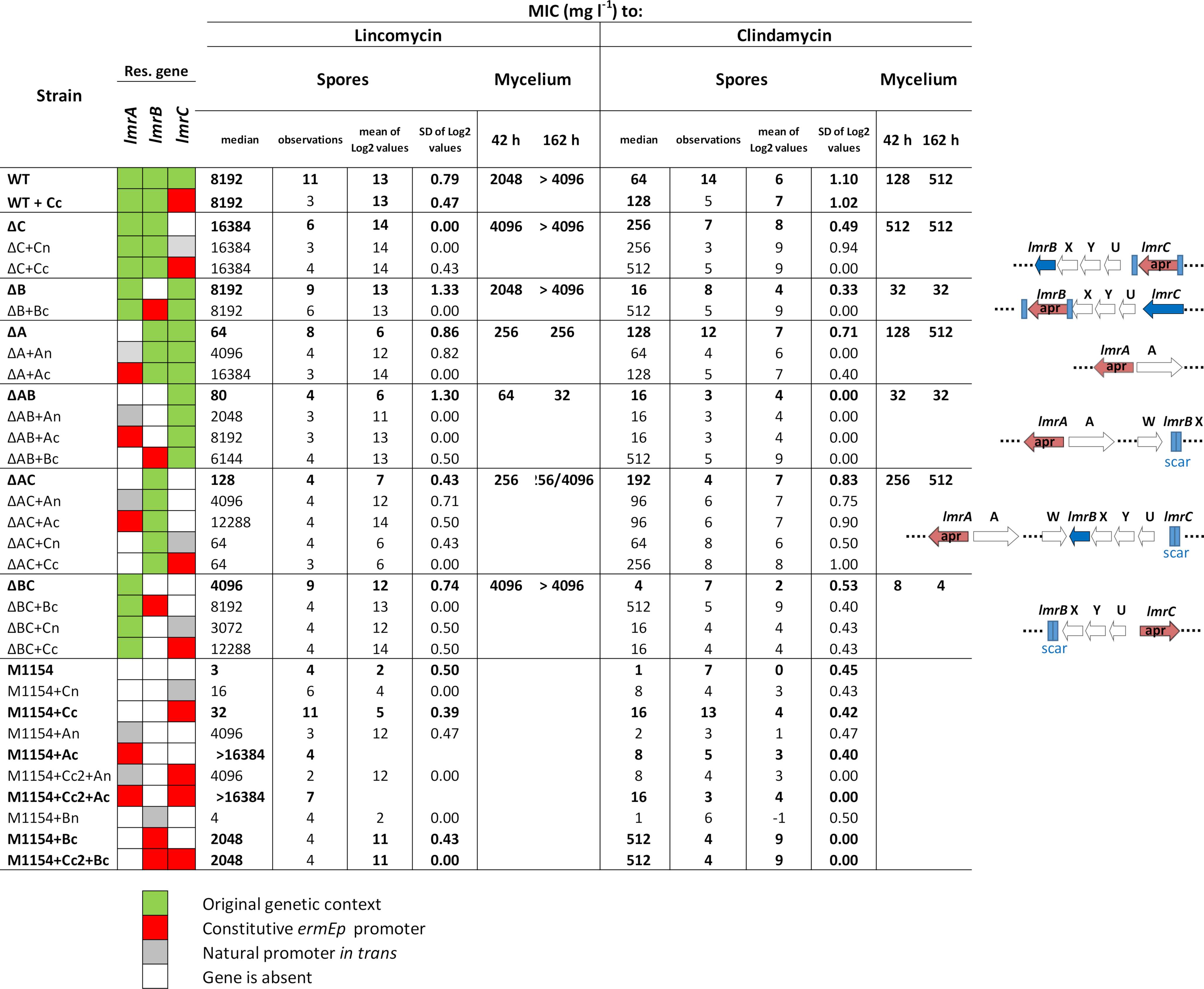

Supplement: FIG S1 [file mbio.01731-21-sf001.tif]

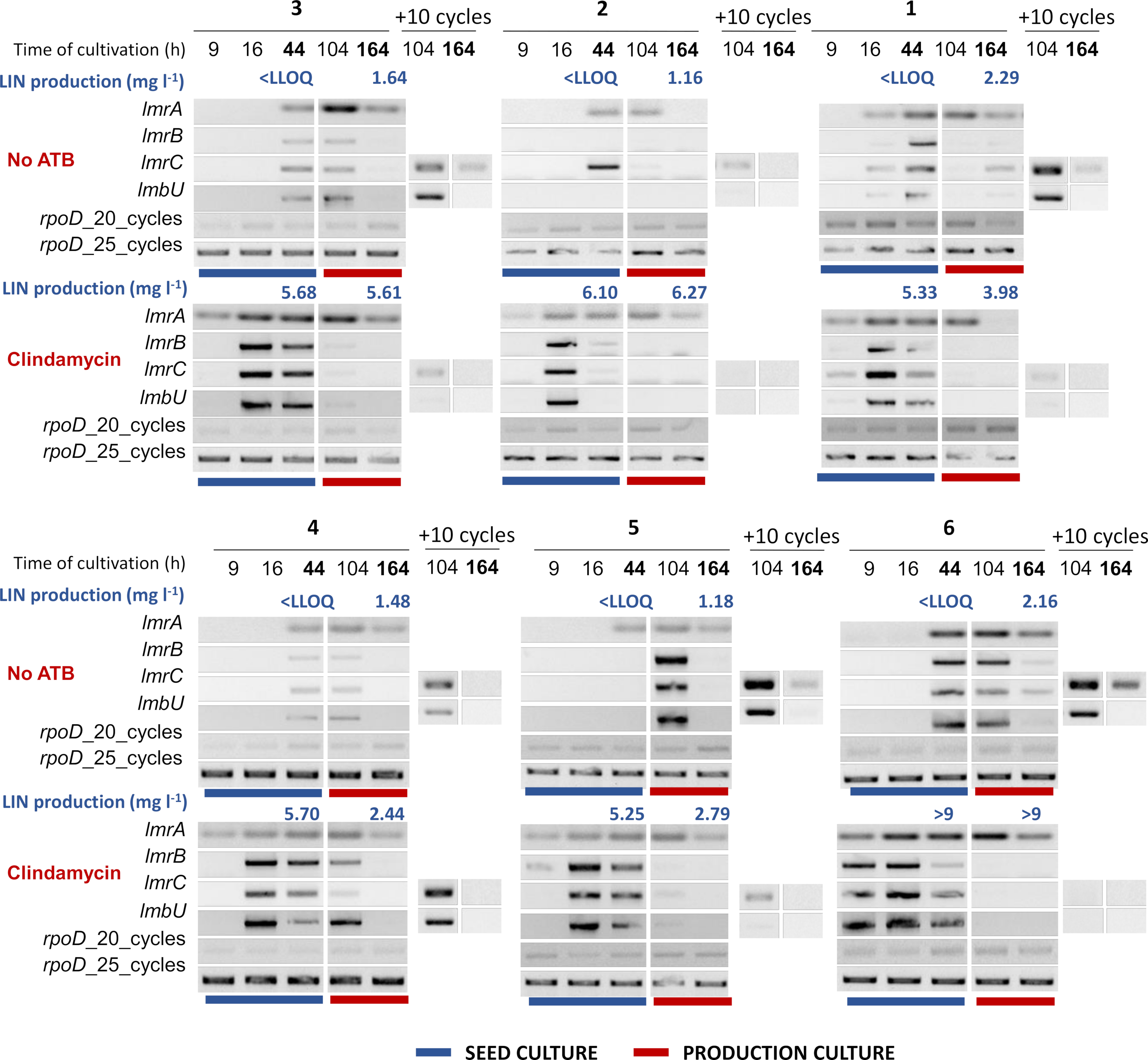

Supplement: FIG S2 [file mbio.01731-21-sf002.tif]

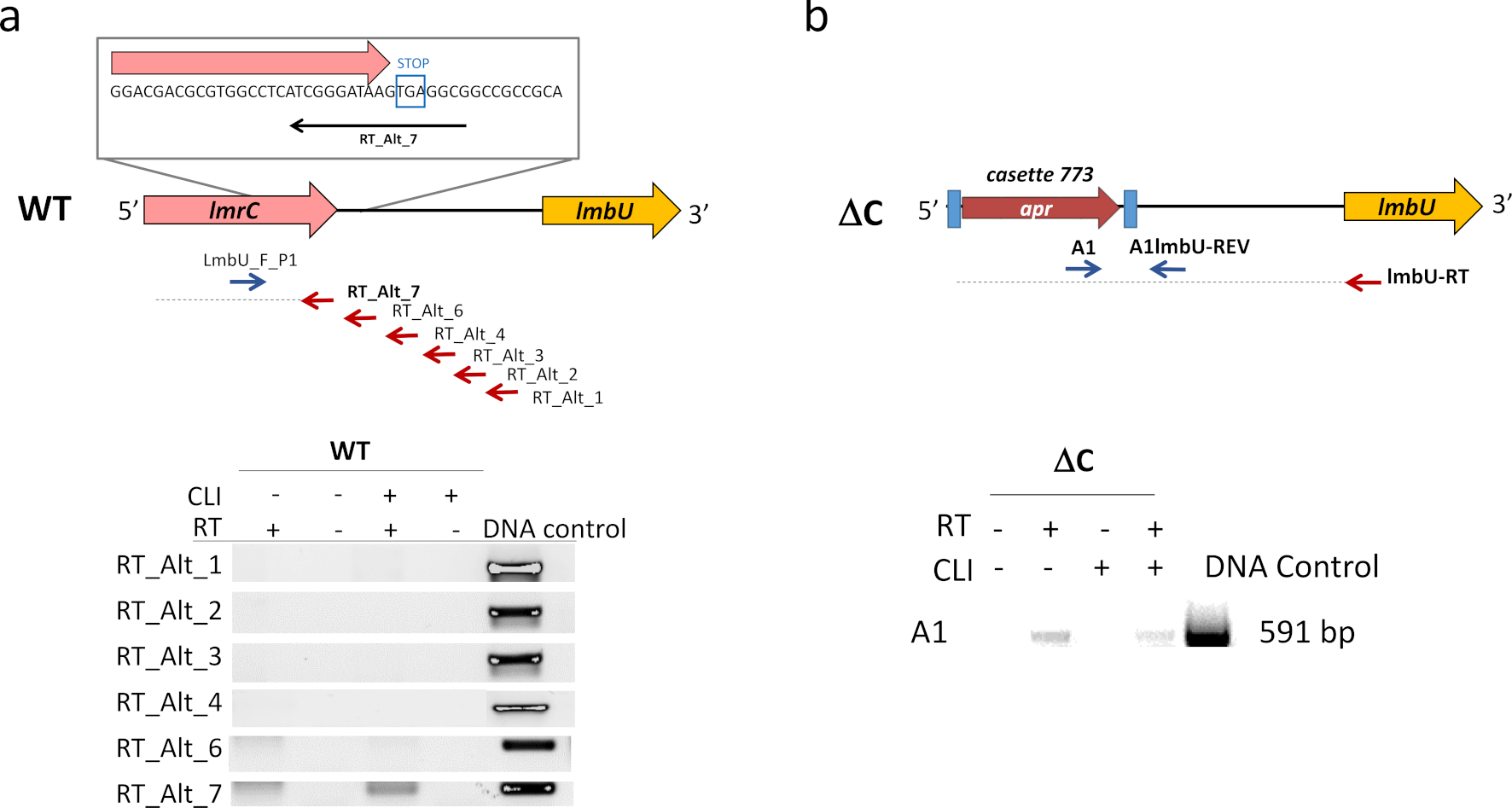

Supplement: FIG S3 [file mbio.01731-21-sf003.tif]

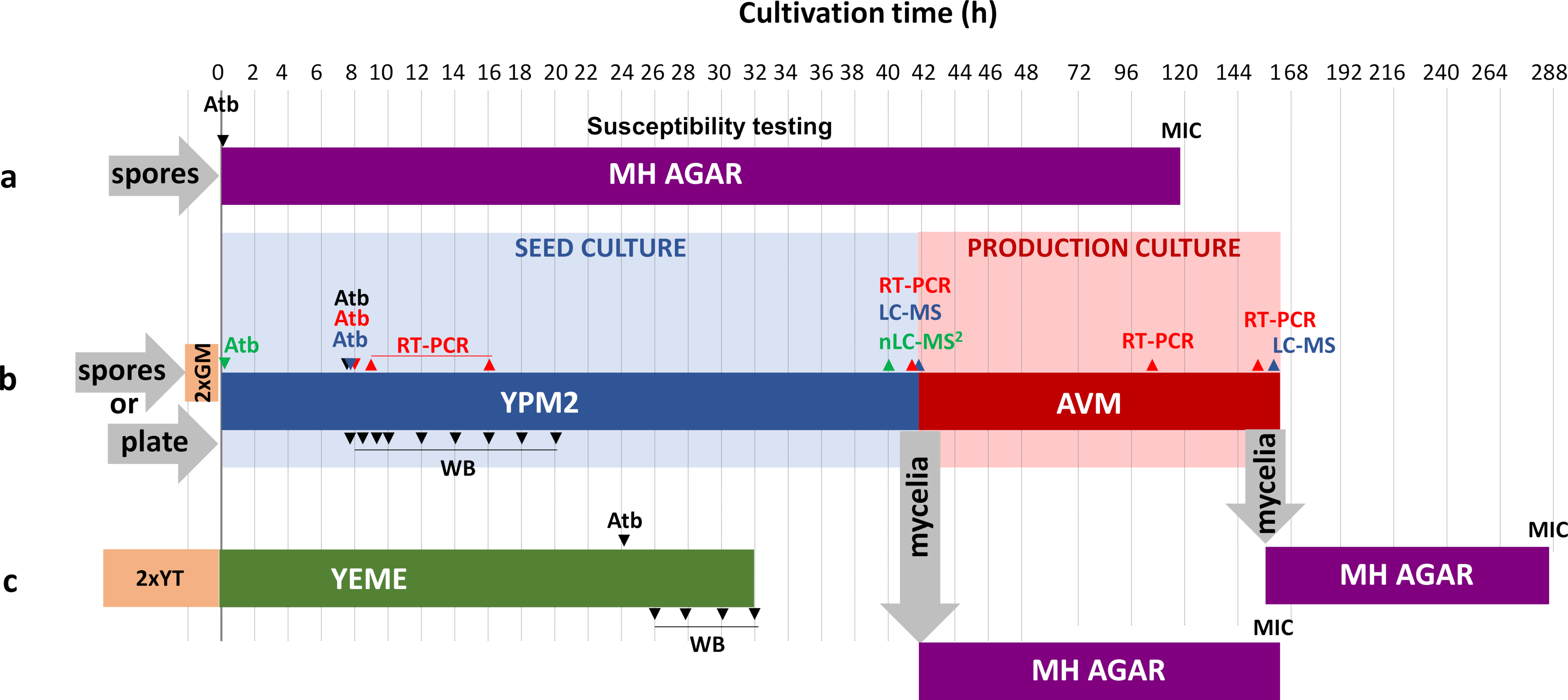

Supplement: FIG S7 [file mbio.01731-21-sf007.tif]

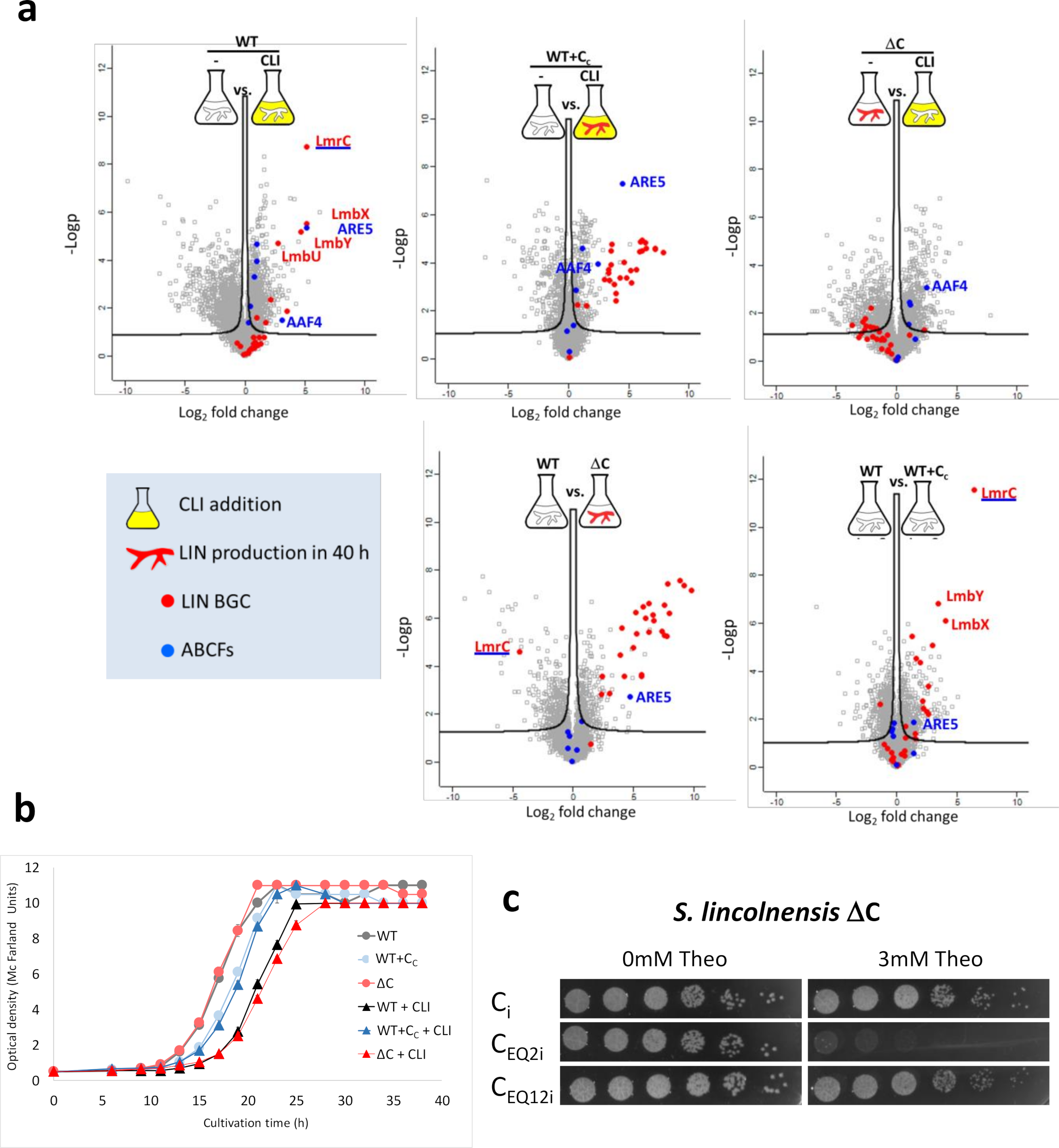

Supplement: FIG S4 [file mbio.01731-21-sf004.tif]

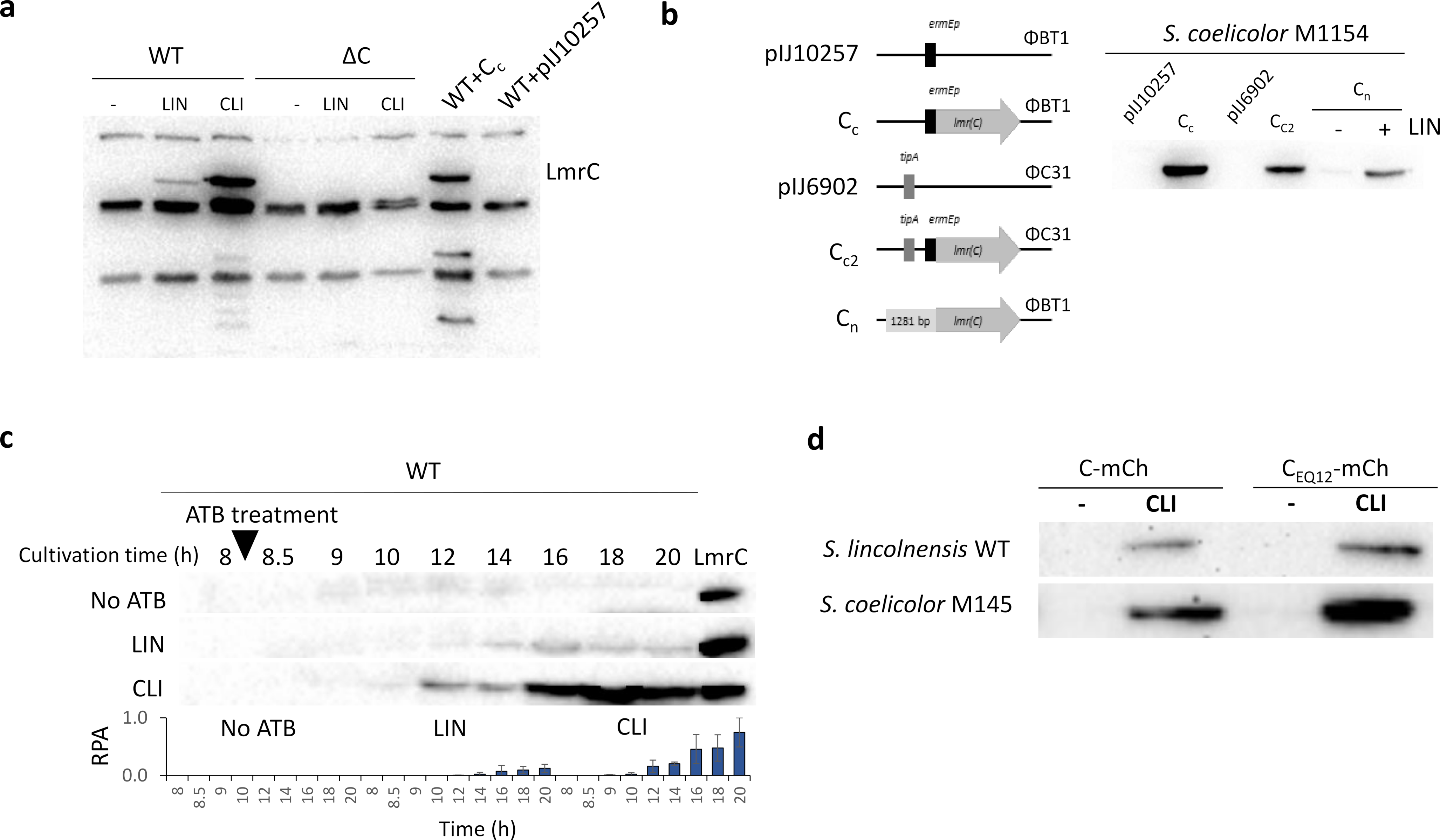

Supplement: FIG S5 [file mbio.01731-21-sf005.tif]

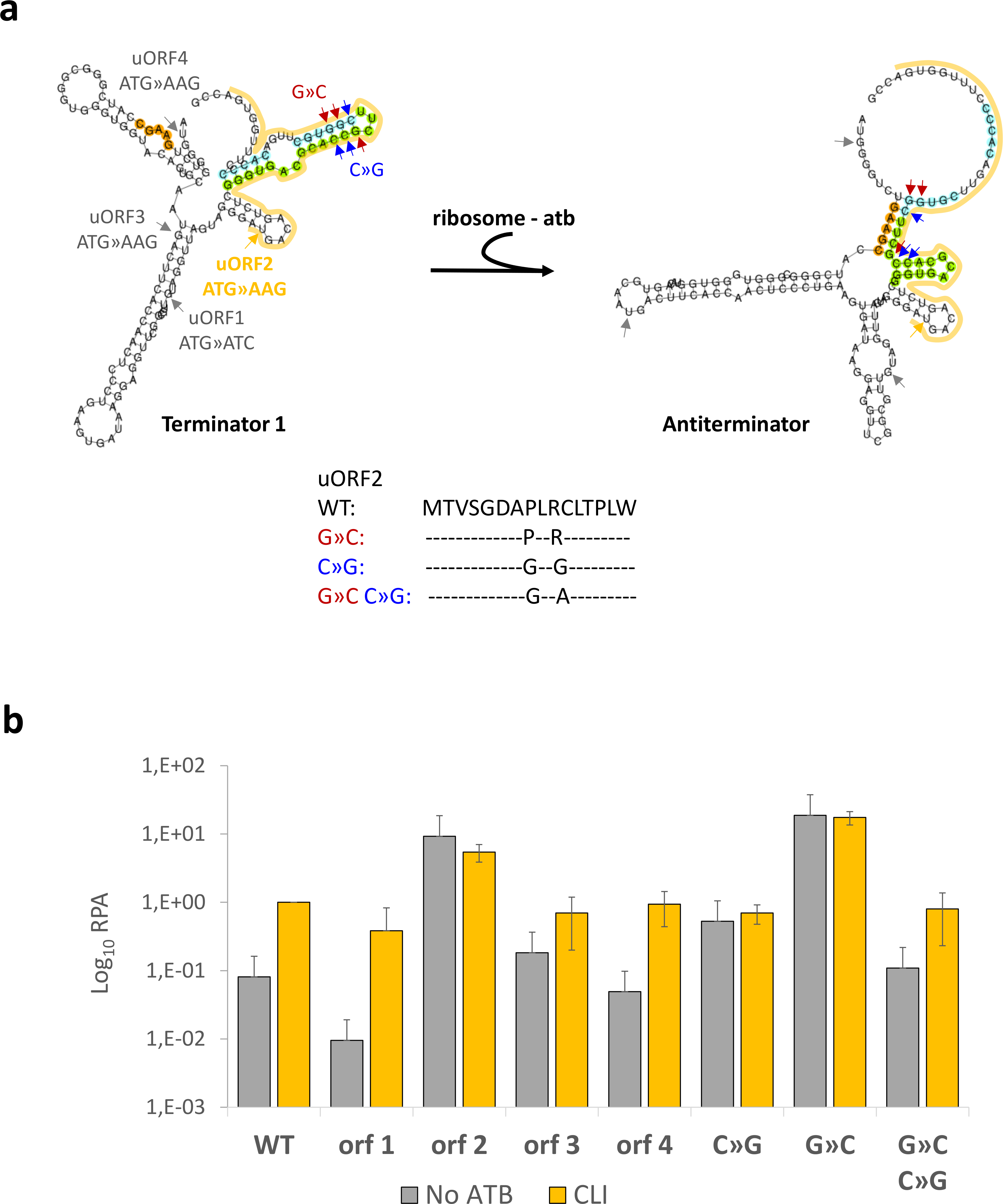

Supplement: FIG S6 [file mbio.01731-21-sf006.tif]
