# Supplementary material for: Beyond Self-Resistance: ABCF ATPase LmrC Is a Signal-Transducing Component of an Antibiotic-Driven Signaling Cascade Accelerating the Onset of Lincomycin Biosynthesis
Source: mBio. 2021 Sep 7;12(5):e01731-21. doi: 10.1128/mBio.01731-21 (PMC8546547; doi:10.1128/mBio.01731-21)
Supplement: TABLE S1 [file mbio.01731-21-st001.pdf]

Antibotic	MIBig or GenBank accession no.	Strain	Antibiotic class	ABCF in BGC	GenBank accession no.	ABCF subfamilly	BGC encoded resistance determinants				BGC encoded regulators		
							methyltransferase	efflux	modification	ribosome recycling	transcriptional regulators	two component system	γ butyrolacton regulation
**Lincomycin**	BGC0000907	*Streptomyces lincolnensis ATCC25466*	lincosamide	LmrC	WP_067425694.1	ARE5		•			•		
**Virginiamycin M**		*Streptomyces virginiae MAFF 10-06014*	macrolide	VarM	BAA96297.1	ARE5		•			•	•	•
**Virginiamycin S1**	BGC0001116		cyclodepsipeptide	VarL	WP_078909286.1	AAF4							
**Lankacidin**	BGC0001100	*Streptomyces rochei 51252*	macrolide	LkcJ	WP_011113090	ARE5		•			•		•
				LkcI	WP_011113091.1	AAF4							
**Lankamycin**	BGC0002033		macrolide	LkmN	BAC76502	ARE4			•				
**Spiramycin**	NZ_CP012382.1	*Streptomyces ambofaciens ATCC 23877*	macrolide	SrmB	WP_053138477.1	ARE4	•			•	•		
**Tylosin**		*Streptomyces fradiae ATCC 10745*	macrolide	TlrC	WP_063856491.1	ARE4	•			•	•		•
**Oleandomycin**		*Streptomyces antibioticus ATCC 11891*	macrolide	OleB	AAA50325.1	ARE4		•	•				
**angolamycin**	BGC0000019	*Streptomyces eurythermus*	macrolide	Orf13*	ABW91161	ARE4	•				•		
**griseoviridin**	BGC0000459	*Streptomyces griseoviridis*	cyclodepsipeptide	SgvT2	AGN74869	ARE4		•			•		•
**tylactone**	BGC0001812	*Micromonospora chalcea subsp. Izumensis*	polyketide	JuvA	ARW71479	ARE4	•				•		
**rosamicin**	BGC0001830	*Salinispora pacifica (mooerana)*	macrolide	Spr3	AKG06371	ARE4					•		
**A201A**	BGC0001138	*Saccharothrix mutabilis s. capreolus NRRL 3817*	aminonucleoside	Ard1	CAA59109.1	AAF1		•	•				
**hygromycin A**	BGC0000699	*Streptomyces hygroscopicus* NRRL 2388	aminoglycoside	Hyg28	ABC42565.1	AAF1	•	•	•		•		
**K252a**	FJ031030.1	*Nonomuraea longicatena* NRRL15532	indolocarbazole	NokW	ACN29728	AAF1		•			•	•	
**amicetin**	BGC0000953	*Streptomyces vinaceusdrappus* NRRL 2363	pyranosyl nucleoside	AmiO	AEF16054	AAF2		•			•		
**mildiomycin**	BGC0000882	*Streptomyces rimofaciens* ZJU5119	peptidyl nucleoside	MilP	AFD20756	AAF2		•	•		•		
**taxlllaid**	BGC0001133	*Xenorhabdus bovienii* SS-2004	lipodepsipeptide	XBJ1_0786	CBJ79927	Uup							
**marformycin**	BGC0001214	*Streptomyces drozdowiczii* SCSIO 10141	cyclodepsipeptide	MfnR	AJV88390	EttA					•		
**thiocillin**	BGC0000612	*Bacillus cereus* ATCC 14579	thiazolylpeptide	TclW	AAP11941	YfmM		•			•		

Organism abbrev.			No. of ABCFs	ABCF subfamilies
**Miau**	NC_014391.1	*Micromonospora aurantiaca* ATCC 27029	7	AAF6, **AAF4**, EttA, **ARE5**, Uup, AAF2, AAF1
**Noco**	ASM37988v1	*Nonomuraea coxensis* DSM 45129	8	EttA, Uup, AAF6, AAF2, ARE4, AAF2, **ARE4, ARE5**
**Saes**	NC_019673.1	*Saccharothrix espanaensis* DSM 44229	11	AAF1, EttA, AAF2, Uup, AAF1, AAF6, **ARE5, AAF4, AAF4, ARE4**, AAF6
**Sapa**	ASM51467v1	*Salinispora pacifica* DSM 45548	5	EttA, AAF2, Uup, AAF6, ARE4
**Stli**	NZ_CP016438.1	*Streptomyces lincolnensis* ATCC 25466	9	**LmrC**, EttA, Uup, AAF6, AAF2, AAF1, **ARE5, AAF4, AAF4**
**Stal**	NC_020990.1	*Streptomyces albidoflavus* (albus) J1074	11	ARE5, ARE4, AAF6, AAF4, AAF4, EttA, ARE4, Uup, AAF2, ARE5, AAF1
**Stav**	NC_003155.5	*Streptomyces avermitilis* MA-4680	6	AAF2, Uup, EttA, **ARE5**, AAF1, AAF6
**Stgr**	NC_010572.1	*Streptomyces griseus subsp. griseus* NBRC 13350	9	**ARE5, ARE4,** AAF2, AAF1, **ARE5**, Uup, EttA, AAF6, **ARE4**
**Sthy**	NC_017765.1	*Streptomyces hygroscopicus subsp. jinggangensis* 5008	8	AAF6, EttA, Uup, **AAF4, ARE5**, AAF2, **ARE5**, AAF1
**Stpr**	NZ_CM000950.1	*Streptomyces pristinaespiralis* ATCC 25486	9	**ARE4**, AAF2, **ARE4, ARE5**, AAF1, Uup, AAF6, **AAF4, ARE5**
**Stri**	ASM33118v1	*Streptomyces rimosus subsp. rimosus* ATCC 10970	10	**AAF4,** AAF1, **ARE5, ARE5**, Uup, **ARE4**, AAF6, AAF2, EttA, AAF6
**Stsv**	NZ_CM000951.1	*Streptomyces sviceus* ATCC 29083	7	AAF6, EttA, Uup, **AAF4, ARE5**, AAF2, **ARE5**
**Stve**	NZ_CP029197.1	*Streptomyces venezuelae* ATCC 10712	8	Uup, **ARE4**, AAF2, EttA, AAF6, **ARE4, ARE5**, AAF1
**Stvi**	ASM72045v1	*Streptomyces virginiae*	6	AAF1, Uup, AAF2, **ARE5**, EttA, **ARE4**
